# UHRF1 deficiency exacerbates intestinal inflammation by epigenetic modulation of NPY1R gene methylation

**DOI:** 10.1172/jci.insight.190894

**Published:** 2026-02-09

**Authors:** Yanan Han, Lina Sun, Yanxing Liu, Xiaohui Zhang, Hao Liu, Haohao Zhang, Xiaoxia Ren, Fenfan Wang, Huafeng Fan, Jie Chen, Dan Liu, Daiming Fan, Yuanyuan Lu, Xue Bai, Ying Fang, Kaichun Wu, Xiaodi Zhao

**Affiliations:** 1 The Affiliated Children’s Hospital of Xi’an Jiaotong University, Xi’an, Shaanxi, China.; 2 State Key Laboratory of Holistic Integrative Management of Gastrointestinal Cancers, Xijing Hospital, Fourth Military Medical University, Xi’an, Shaanxi, China.; 3 Department of Gastroenterology, Tangdu Hospital, Fourth Military Medical University, Xi’an, Shaanxi, China.

**Keywords:** Gastroenterology, Inflammation, Epigenetics

## Abstract

Epigenetic modifications play a crucial role in the pathogenesis of inflammatory bowel disease (IBD) by mediating gene-environment interactions. We previously showed that UHRF1, a central regulator of DNA methylation, contributes to cancer progression; however, its function in IBD remains poorly understood. Here, we revealed that UHRF1 was frequently reduced in inflamed tissues of patients with IBD and that its deficiency exacerbated intestinal epithelial cell (IEC) damage. Through a multilevel approach incorporating human cell models and an intestinal epithelial-specific *Uhrf1*-KO mouse model, we established UHRF1 as a key mitigator of IBD progression. Mechanistically, UHRF1 bound to the NPY1R promoter, promoted its methylation, and led to transcriptional suppression. The NPY1R upregulation resulting from UHRF1 deficiency attenuated cAMP/PKA/CREB signaling in IECs, thereby enhancing NF-κB activation and subsequent proinflammatory responses, which compromised intestinal epithelial barrier integrity. Furthermore, we identified miR-141 as a negative regulator of NPY1R, highlighting its potential as a therapeutic agent. Collectively, our results identified the UHRF1/NPY1R regulatory axis as a critical epigenetic mechanism in intestinal inflammation and underscored its dual promise for IBD diagnostics and therapy.

## Abstract 2

UHRF1 shows potential as a novel diagnostic tool for IBD patients and lays the groundwork for developing microRNA-based therapeutic strategies targeting UHRF1 in IBD treatment.

## Graphical abstract

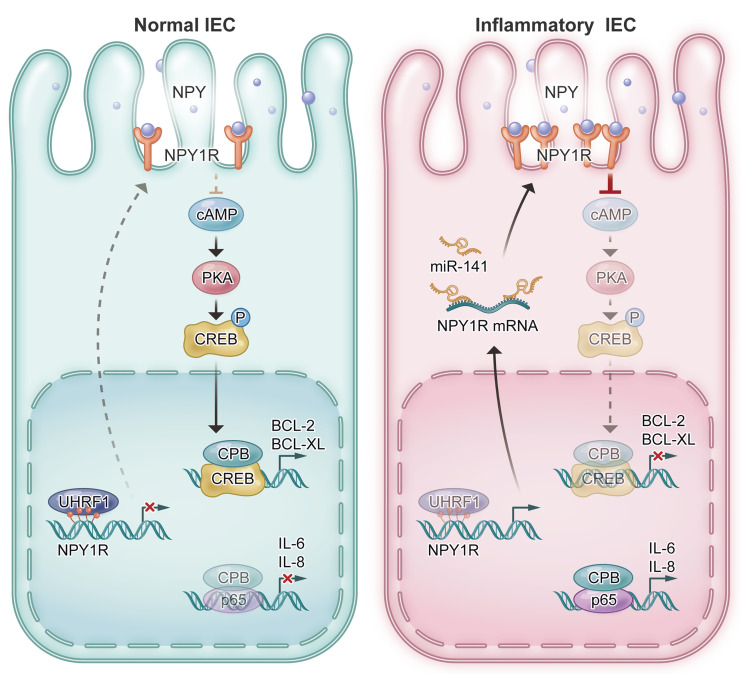

## Introduction

Inflammatory bowel disease (IBD) is a chronic inflammatory disorder of the gastrointestinal tract, characterized by a relapsing and remitting manner. It primarily encompasses 2 subtypes: ulcerative colitis (UC) and Crohn’s disease (CD). The onset of IBD involves a complex interplay of genetic, environmental, and immune dysregulation factors; however, its precise etiology and pathogenesis remain poorly understood (1, 2). Therefore, exploring the underlying pathogenic mechanisms of IBD and identifying novel therapeutic targets are crucial for advancing clinical treatment.

Growing evidence indicates that epigenetic modifications are associated with the pathogenesis of IBD (3, 4). These alterations represent crucial molecular mechanisms in IBD progression, influencing immune regulation, intestinal epithelial barrier integrity, and autophagy (5). Epigenetic modifications primarily include DNA methylation, histone methylation, histone acetylation, RNA modifications, and noncoding RNA. Among these, DNA methylation stands as one of the most stable epigenetic modifications in vertebrates, where it plays a critical role in gene regulation (6). Genes linked to IBD may undergo marked changes in expression due to alterations in methylation status, thereby affecting disease onset and progression (7, 8). For instance, DNA hypomethylation of *Zbtb7b* promotes CD4^+^ T cell maturation, thereby driving inflammatory cytokine production and contributing to colonic inflammation in UC (9). Under inflammation conditions, DNA hypomethylation of *EBI3* can promote the formation of the antiinflammatory cytokine IL-35, highlighting its potential as a therapeutic target in IBD (10). However, the key regulatory factors that drive DNA methylation changes in IBD and their critical downstream gene targets remain largely unexplored. Unraveling these mechanisms is essential for deciphering the epigenetic basis of IBD and developing novel therapeutic strategies.

Ubiquitin-like with PHD and RING finger domains 1 (UHRF1) is a key epigenetic regulator and one of the few proteins capable of simultaneously recognizing both histone and DNA modifications, playing an essential role in maintaining DNA methylation by recruiting DNA methyltransferase 1 (DNMT1) to replication forks (11). UHRF1 regulates de novo DNA methylation through recruitment of de novo DNA methyltransferases DNMT3A and DNMT3B to chromatin, enabling these enzymes to establish DNA methylation sites (12). UHRF1 has been implicated in IBD progression, primarily through its immunomodulatory functions. In colonic Tregs, UHRF1 represses the gene *Cdkn1a* via promoter methylation, thereby maintaining their proliferation and functional maturation to suppress excessive immune responses during murine colitis (13). In macrophages, UHRF1-mediated DNA methylation controls the expression of TNF-α, shaping the inflammatory response in experimental colitis models (14). The intestinal epithelium forms a critical barrier protecting the organism from microbes and other proinflammatory stimuli (15). Although a study in zebrafish revealed that loss of *Uhrf1* reduces *Tnfa* promoter methylation in intestinal epithelial cells (IEC), leading to barrier dysfunction and inflammation (16), our previous work identified UHRF1 overexpression in gastric cancer (17). The expression pattern, functional role, and mechanistic contribution of UHRF1 within human IECs remain poorly elucidated. In this study, we demonstrate that UHRF1 expression is reduced in the intestinal epithelium of patients with IBD and reveal its protective role using clinical samples and experimental colitis models. We further elucidate its molecular mechanism by showing that UHRF1 deficiency induces hypomethylation of NPY1R, thereby activating NF-κB–mediated inflammation. These findings identify the UHRF1/NPY1R axis as a promising target for novel diagnostic and therapeutic strategies in IBD.

## Results

### UHRF1 is downregulated in IECs under inflammatory conditions.

**Figure 1:**
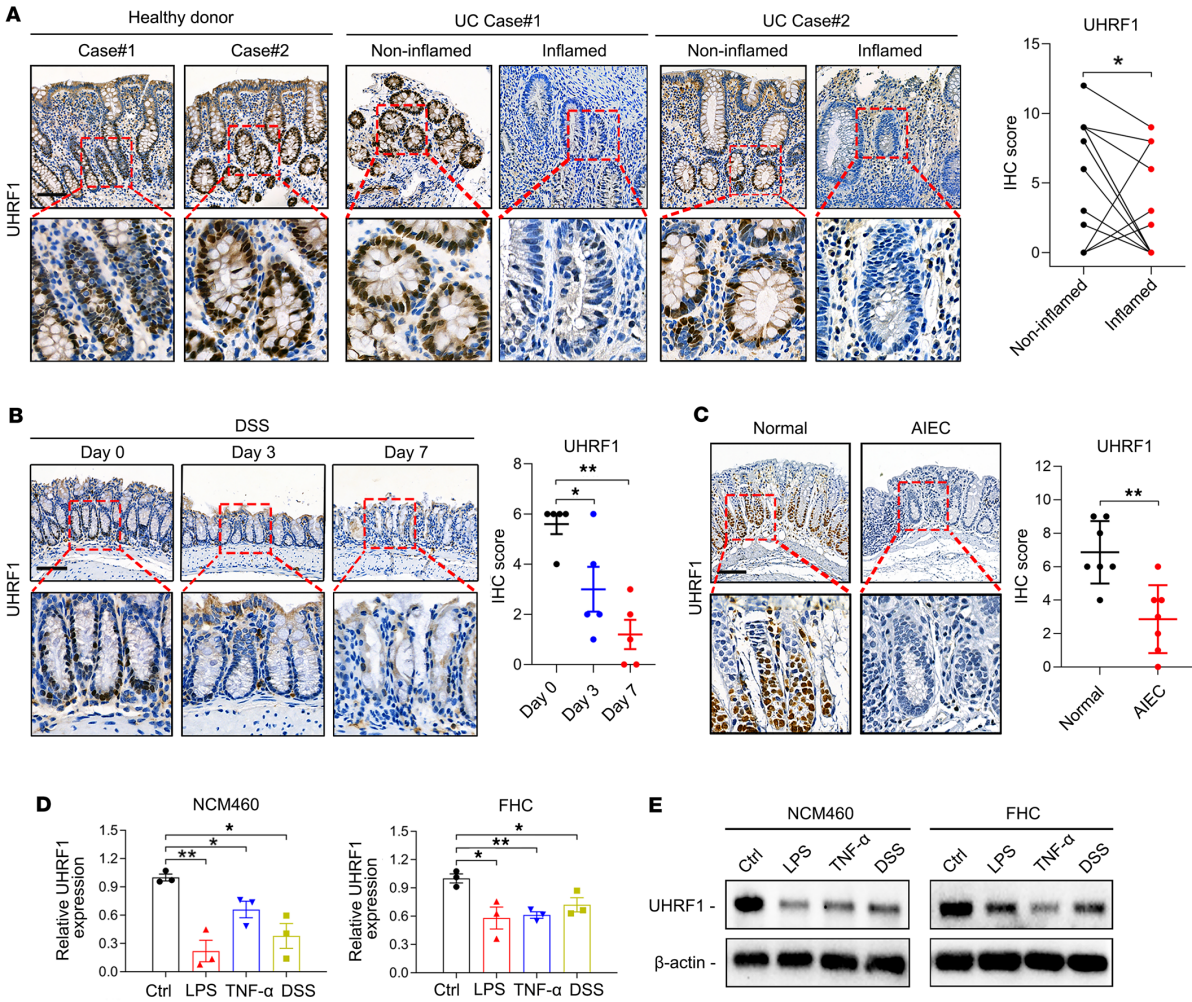
UHRF1 is downregulated in IECs under inflammatory conditions. (**A**) Representative staining images and quantification of UHRF1 in the indicated tissues from healthy donors and patients with UC. Scale bar: 50 μm. (**B**) Representative staining images and quantification of UHRF1 in colon tissues from mice treated with DSS and sacrificed at different time points (*n* = 5). Scale bar: 50 μm. (**C**) Representative staining images and quantification of UHRF1 in colon tissues from mice treated with AIEC or controls (*n* = 7). Scale bar: 50 μm. (**D** and **E**) mRNA (**D**) and protein (**E**) expression of UHRF1 in NCM460 and FHC cells treated with LPS, TNF-α, or DSS. *P* values were determined by 2-tailed Student’s *t* tests (**A** and **C**) or 1-way ANOVA (**B** and **D**). Data are shown as mean ± SD. *P < 0.05, **P < 0.01.

To establish the clinical relevance of UHRF1 in IBD, we measured UHRF1 expression in patients with UC. Our analysis included paired inflamed and noninflamed mucosal samples from 15 patients with active UC, inflamed tissues from 10 independent patients with UC, and normal colonic tissues from 25 healthy donors (Supplemental Table 1; supplemental material available online with this article; https://doi.org/10.1172/jci.insight.190894DS1). IHC revealed strong nuclear UHRF1 expression in IECs of healthy donors and noninflamed UC mucosa, whereas a marked reduction was observed in inflamed UC tissues (Figure 1A and Supplemental Figure 1A). Ki-67 staining revealed no significant difference between inflamed and noninflamed tissues from patients with UC, suggesting that UHRF1 suppression is independent of proliferative status (Supplemental Figure 1B). Next, we assessed UHRF1 expression patterns across multiple mouse models of experimental intestinal inflammation. In the DSS-induced acute colitis model, characterized by colonic shortening and elevated inflammation scores (Supplemental Figure 1, C and D), IHC and Western blot analyses revealed a progressive time-dependent decrease in UHRF1 expression in colonic tissues on days 3 and 7 (Figure 1B and Supplemental Figure 1E). Consistently, in a model of adherent-invasive *E*. *coli* (AIEC) infection, UHRF1 expression was also significantly reduced compared with the control group (Figure 1C). Furthermore, to validate our observations in human contexts, we established in vitro inflammation models using the human IEC lines NCM460 and FHC. Stimulation with specific inflammatory mediators, including the microbial infection factor LPS, the chemical agent DSS, and the immunological inflammatory cytokine TNF-α, consistently suppressed UHRF1 expression in both NCM460 and FHC cells (Figure 1, D and E). Notably, this reduction in UHRF1 was also observed in serum-starved, cell cycle–arrested NCM460 cells (Supplemental Figure 1F), indicating that inflammatory stimuli suppress UHRF1 independently of cell cycle progression. These results establish that UHRF1 downregulation is a consistent response of IECs to inflammatory stress.

### UHRF1 deficiency sensitize IECs to inflammatory damage.

**Figure 2:**
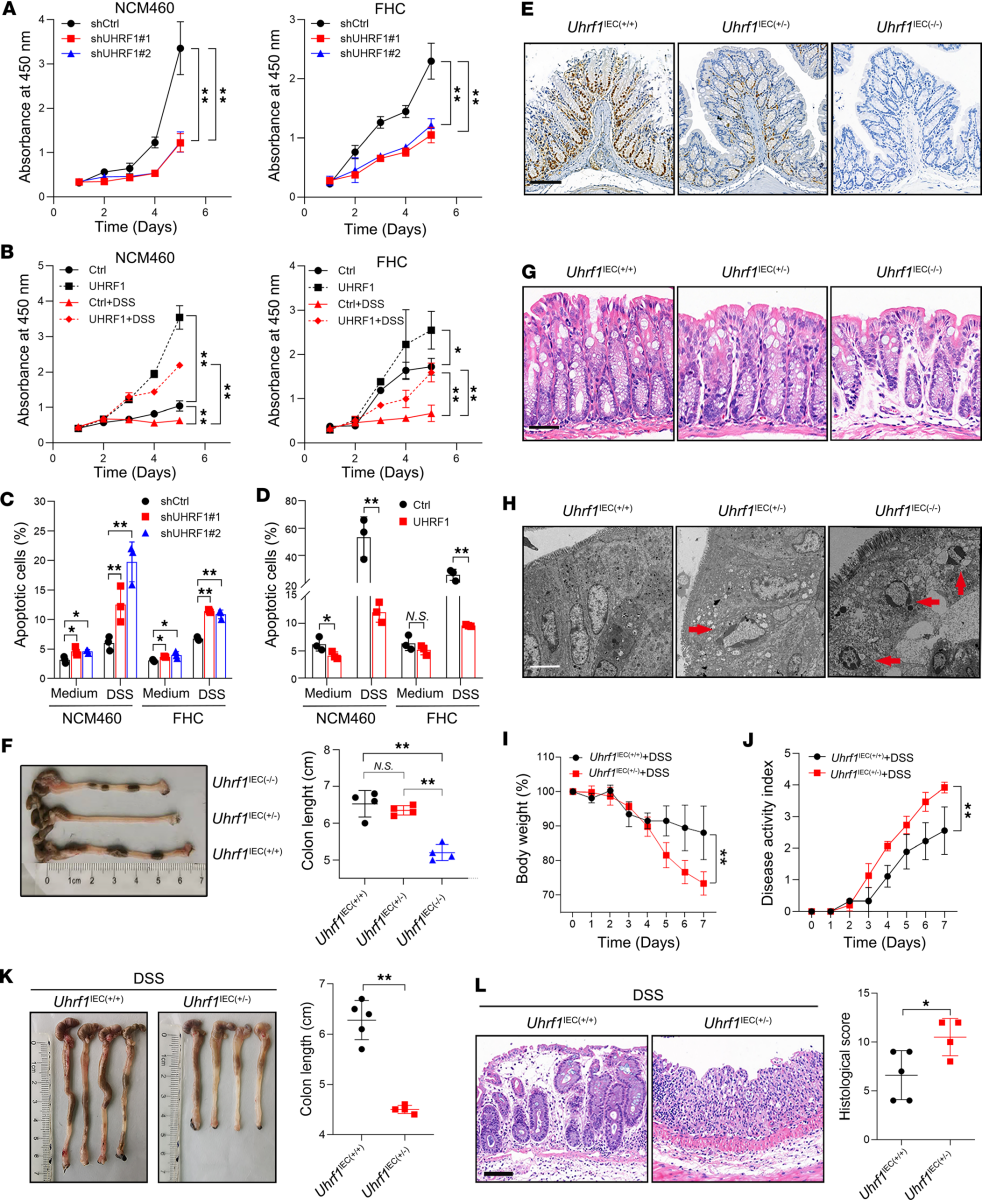
UHRF1 deficiency sensitize IECs to inflammatory damage. (**A**) Proliferation of NCM460 and FHC cells infected with shUHRF1 or shCtrl. (**B**) Proliferation of NCM460 and FHC cells infected with UHRF1-overexpressing (UHRF1) vector or empty control (Ctrl) vector and treated with or without DSS. (**C**) Apoptotic rates of NCM460 and FHC cells with shUHRF1 or shCtrl and treated with or without DSS.(**D**) Apoptotic rates of NCM460 and FHC cells with UHRF1 or Ctrl and treated with or without DSS. (**E**) Representative staining images of UHRF1 in colonic tissues from *Uhrf1*^IEC(+/+)^, *Uhrf1*^IEC(+/–)^ and *Uhrf1*^IEC(–/–)^ mice. Scale bar: 100 μm. (**F**) Representative image and quantification of colon length from *Uhrf1*^IEC(+/+)^, *Uhrf1*^IEC(+/–)^ and *Uhrf1*^IEC(–/–)^ mice (*n* = 4). (**G**) Representative H&E-stained colon tissues from *Uhrf1*^IEC(+/+)^, *Uhrf1*^IEC(+/–)^ and *Uhrf1*^IEC(–/–)^ mice. Scale bar: 50 μm. (**H**) Representative images of IECs from *Uhrf1*^IEC(+/+)^, *Uhrf1*^IEC(+/–)^, and *Uhrf1*^IEC(–/–)^ mice by transmission electron microscopy. Arrows indicate typical apoptotic ultrastructural characteristics present in apoptosis. Scale bar: 5 μm. (**I** and **J**) Body weight curve (**I**) and disease activity index (**J**) of *Uhrf1*^IEC(+/+)^ and *Uhrf1*^IEC(+/−)^ mice treated with DSS for 7 days (*n* = 5). (**K**) Colon length of *Uhrf1*^IEC(+/+)^ and *Uhrf1*^IEC(+/−)^ mice sacrificed at the end of DSS-induced colitis model (*n* = 5). (**L**) Representative H&E staining and histological score of colon tissues from *Uhrf1*^IEC(+/+)^ and *Uhrf1*^IEC(+/−)^ mice (*n* = 5). Scale bar: 100 μm. *P* values were determined by 2-way ANOVA (**A**, **B**, **I**, and **J**), 1-way ANOVA (**C** and **F**), or 2-tailed Student’s *t* tests (**D**, **K**, and **L**). Data are shown as mean ± SD. *P < 0.05, **P < 0.01.

To investigate the functional role of UHRF1 in IECs, we established stable UHRF1-overexpression and knockdown models in NCM460 and FHC cell lines using lentiviral transduction (Supplemental Figure 2, A and B). Cell apoptosis assay and Cell Counting Kit 8 (CCK-8) assays revealed that UHRF1 knockdown suppressed proliferation in both NCM460 and FHC cells (Figure 2A). Notably, UHRF1 overexpression enhanced proliferation and rescued the DSS-induced impairment of cell growth in both cell lines (Figure 2B). Flow cytometry analysis revealed that UHRF1 knockdown promoted apoptosis compared with controls (Figure 2C and Supplemental Figure 2C). Conversely, UHRF1 overexpression not only reduced baseline apoptosis but also rescued DSS-induced apoptosis in both cell lines (Figure 2D and Supplemental Figure 2D). These results establish that UHRF1 enhances cell viability and suppresses apoptosis in IECs under inflammatory conditions in vitro.

To investigate the in vivo function of UHRF1, we generated IEC-specific *Uhrf1*-KO mice by crossing *Uhrf1*^fl/fl^ mice with *Villin-Cre* mice (Supplemental Figure 2E). IHC analysis confirmed that UHRF1 expression was predominantly localized to the colonic epithelium, with reduced levels in *Uhrf1*^IEC(+/−)^ mice and complete absence in *Uhrf1*^IEC(−/−)^ mice (Figure 2E). Compared with WT littermates, *Uhrf1*^IEC(+/−)^ mice exhibited moderate colonic shortening, whereas *Uhrf1*^IEC(−/−)^ mice showed a more severe reduction in colon length (Figure 2F). Histological examination revealed that *Uhrf1*^IEC(+/−)^ mice developed mild villus shortening and crypt distortion compared with WT mice, while *Uhrf1*^IEC(−/−)^ mice exhibited more pronounced villus atrophy and disrupted crypt architecture (Figure 2G). Notably, IECs from both *Uhrf1*^IEC(+/−)^ and *Uhrf1*^IEC(−/−)^ mice exhibited pronounced nuclear fragmentation (Figure 2H), consistent with UHRF1 deficiency promoting cellular apoptosis. Due to early mortality and non-Mendelian segregation in *Uhrf1*^IEC(−/−)^ mice, we utilized *Uhrf1*^IEC(+/−)^ mice for further experimental analysis. To investigate the protective role of UHRF1 in IBD, we subjected WT and *Uhrf1*^IEC(+/−)^ mice to DSS-induced colitis. Compared with WT littermates, *Uhrf1*^IEC(+/−)^ mice displayed aggravated intestinal pathology, manifested by greater weight loss, elevated disease activity index (DAI) scores, pronounced colonic shortening, and severe crypt disruption (Figure 2, I–L). These findings indicate that UHRF1 deficiency enhances the susceptibility of IECs to inflammatory damage.

### UHRF1 epigenetically represses NPY1R expression through promoter methylation in IECs.

**Figure 3:**
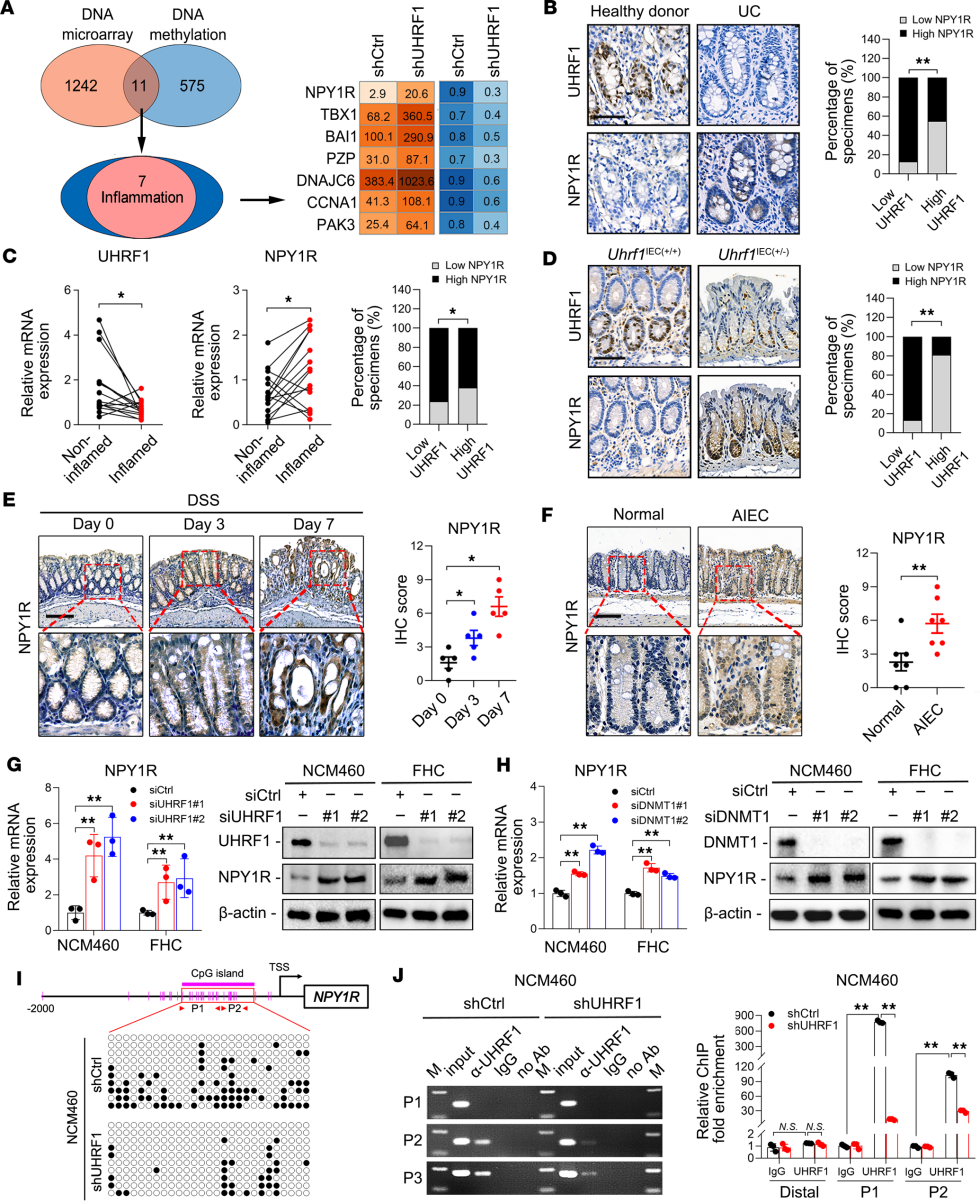
UHRF1 represses NPY1R expression through promoter methylation in IECs. (**A**) Venn diagram for upregulated and hypomethylated genes in NCM460 cells infected with shUHRF1 or shCtrl, and Gene Ontology (GO) enrichment analysis of genes common to these 2 gene sets. (**B**) Representative staining images and correlation analysis of UHRF1 and NPY1R expression in colonic tissues from patients with UC and healthy donors (*n* = 25). Scale bar: 50 μm. (**C**) mRNA expression and correlation analysis of UHRF1 and NPY1R in paired inflamed and noninflamed colon tissue samples from patients with UC (*n* = 15). (**D**) Representative staining images and correlation analysis of UHRF1 and NPY1R in *Uhrf1*^IEC(+/+)^ (*n* = 16) an*d Uhrf1*^IEC(+/−)^ (*n* = 15) mice. Scale bar: 50 μm. (**E**) Representative staining images and quantification of NPY1R in colon tissues from mice treated with DSS and sacrificed at different time points (*n* = 5). Scale bar: 50 μm. (**F**) Representative staining images and quantification of NPY1R in colon tissues from mice treated with AIEC or controls (*n* = 7). Scale bar: 50 μm. (**G**) mRNA and protein expression of NPY1R in NCM460 and FHC cells transfected with siUHRF1 or siCtrl. (**H**) mRNA and protein expression of NPY1R in NCM460 and FHC cells transfected with siDNMT1 or siCtrl. (**I**) Upper: Schematic depicts the CpG island (purple bar) and CpG sites (purple ticks) in the NPY1R promoter. Lower: Methylation status of specific CpG sites was assessed by bisulfite cloning and sequencing. (**J**) Analysis of ChIP-enriched DNA from the NPY1R promoter CpG island by conventional PCR (left) and qPCR (right) in NCM460 cells infected with shUHRF1 or shCtrl. *P* values were determined by χ^2^ test (**B**, **C** [right], and **D**), 1-way ANOVA (**E**, **G**, **H**, and **J**), or 2-tailed Student’s *t* tests (**C** [left and middle] and **F**). Data are shown as mean ± SD. *P < 0.05, **P < 0.01.

To elucidate how UHRF1 exerts its protective effects, we investigated its functional mechanism. Given its central role in DNA methylation (11), we integrated transcriptomic and methylomic analyses to identify downstream epigenetic targets of UHRF1 (Supplemental Figure 3A). Gene Ontology (GO) analysis of inflammation-associated terms identified 7 candidate genes exhibiting concomitant upregulation of expression and reduction in DNA methylation, among which NPY1R showed the most considerable upregulation (Figure 3A). IHC analysis revealed higher NPY1R expression in UC tissues compared with healthy controls, showing an inverse correlation with UHRF1 levels (Figure 3B). Moreover, in paired samples from patients with active UC, inflamed mucosa demonstrated decreased UHRF1 and increased NPY1R expression relative to their noninflamed counterparts (Figure 3C). Consistent with the human data, *Uhrf1*^IEC(+/−)^ mice exhibited elevated NPY1R expression compared with WT littermates, confirming an inverse correlation with UHRF1 (Figure 3D). Furthermore, in experimental colitis models, NPY1R expression increased in a time-dependent manner following DSS challenge (Figure 3E) and was significantly upregulated in mice infected with AIEC (Figure 3F). In line with in vivo findings, NPY1R expression was also markedly induced in both NCM460 and FHC cells upon exposure to LPS, DSS, or TNF-α stimuli (Supplemental Figure 3B). UHRF1 knockdown further augmented NPY1R expression in both NCM460 and FHC cell lines (Figure 3G), confirming its role as a key regulator of NPY1R.

To investigate the mechanism underlying UHRF1-mediated suppression of NPY1R, we considered its established role in maintenance DNA methylation via recruitment of DNMT1 to replication foci (18–20). We therefore examined whether UHRF1 regulates NPY1R in a DNMT1-dependent manner. As anticipated, DNMT1 knockdown in NCM460 and FHC cells markedly upregulated NPY1R expression (Figure 3H). Given the predominant expression of NPY1R in the central nervous system (21), we asked if its expression in colonic tissues is regulated by DNA methylation–based silencing, similar to the oxytocin receptor gene promoter (22). We therefore hypothesized that UHRF1 directly regulates the methylation status of the NPY1R promoter. To test this, we performed bisulfite sequencing in NCM460 cells following UHRF1 knockdown. The results confirmed a significant reduction in DNA methylation at multiple CpG sites within the promoter CpG island (Figure 3I). Furthermore, chromatin immunoprecipitation (ChIP) assays demonstrated direct binding of UHRF1 to the NPY1R promoter (Figure 3J). Collectively, these findings indicate that UHRF1 binds to the NPY1R promoter, induces CpG hypermethylation, and transcriptionally suppresses NPY1R, thereby establishing it as a key downstream effector of UHRF1-mediated epigenetic silencing.

### NPY1R activation antagonizes UHRF1-mediated protection in IECs.

**Figure 4:**
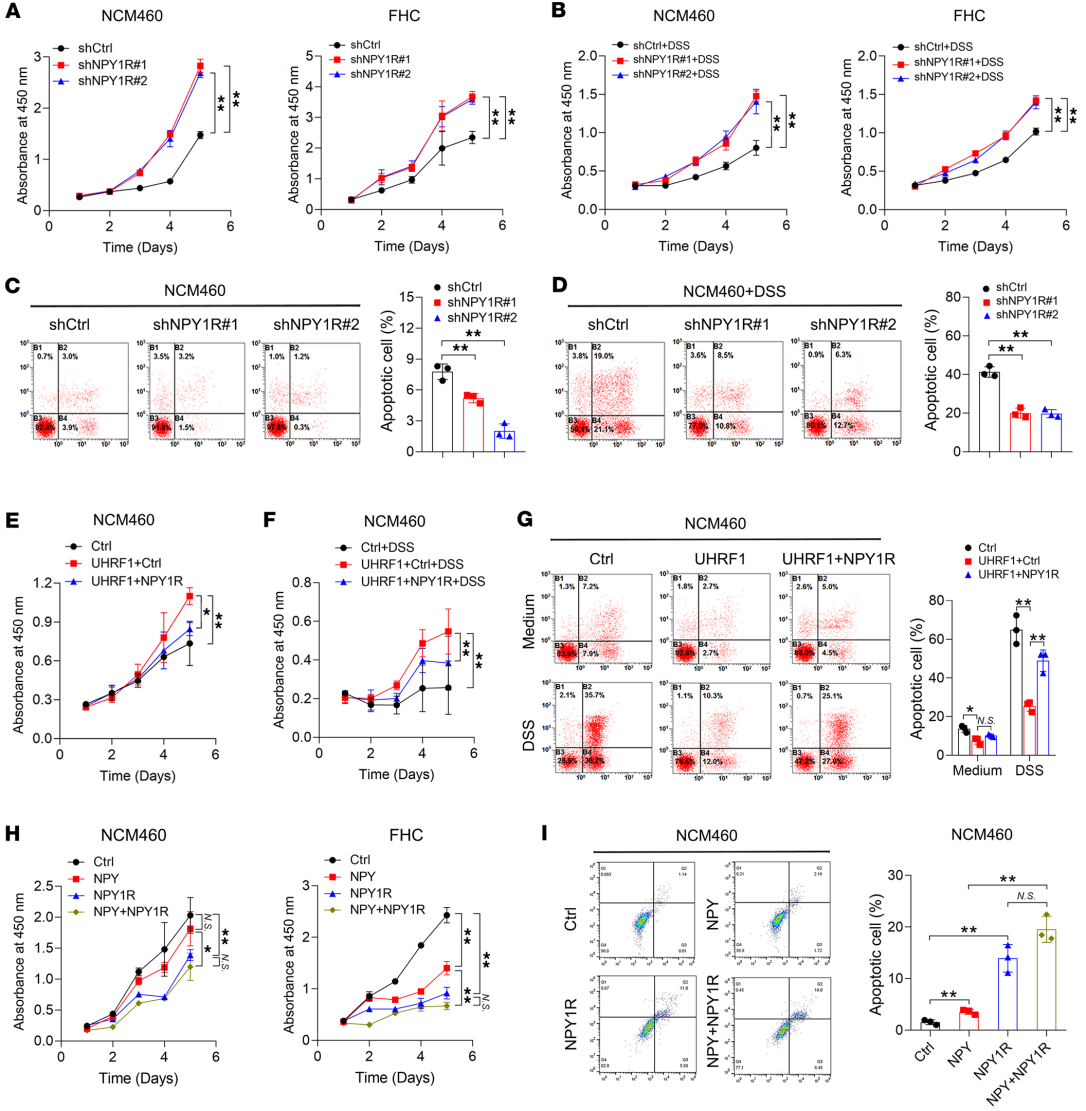
NPY1R activation antagonizes UHRF1-mediated protection in IECs. (**A**) Proliferation of NCM460 and FHC cells infected with shNPY1R or shCtrl. (**B**) Proliferation of NCM460 and FHC cells infected with shNPY1R or shCtrl and treated with DSS. (**C**) Apoptotic rates of NCM460 cells infected with shNPY1R or shCtrl. (**D**) Apoptotic rates of NCM460 cells infected with shNPY1R or shCtrl and treated with or without DSS (**E**) Proliferation of UHRF1-overexpressing NCM460 cells, with or without concurrent NPY1R overexpression. (**F**) Proliferation of UHRF1-overexpressing NCM460 cells with or without concurrent NPY1R overexpression under DSS treatment. (**G**) Apoptotic rates of UHRF1-overexpressing NCM460 cells, with or without concurrent NPY1R overexpression, and treated with or without DSS. (**H**) Proliferation of NCM460 and FHC cells treated with NPY, transfected with NPY1R-overexpressing plasmid, or transfected with the plasmid and treated with NPY. (**I**) Apoptotic rates of NCM460 cells treated with NPY, transfected with NPY1R-overexpressing plasmid, or transfected with the plasmid and treated with NPY. *P* values were determined by 2-way ANOVA (**A**, **B**, **E**, **F**, and **H**) or 1-way ANOVA (**C**, **D**, **G**, and **I**). Data are shown as mean ± SD. *P < 0.05, **P < 0.01.

To validate the function of NPY1R in IECs, we established NPY1R-overexpression and knockdown models in NCM460 and FHC cells (Supplemental Figure 4, A and B). In vitro analysis demonstrated that NPY1R overexpression suppressed proliferation in NCM460 and FHC cells (Supplemental Figure 4, C and D), whereas NPY1R knockdown enhanced proliferation both under basal and DSS-induced inflammatory conditions (Figure 4, A and B). Consistently, NPY1R knockdown attenuated apoptosis in NCM460 and FHC cells under identical experimental settings (Figure 4, C and D). We then investigated whether UHRF1 regulates IECs via NPY1R suppression. In UHRF1-overexpressing NCM460 cells, NPY1R expression counteracted the protective effects of UHRF1 on cell proliferation and apoptosis, both under baseline and DSS-induced inflammatory conditions (Figure 4, E–G). As a G protein–coupled receptor (GPCR), NPY1R functions through binding its ligand neuropeptide Y (NPY), which has been reported to be elevated in the plasma of patients with IBD and implicated in disease-related inflammation (23). We therefore investigated the functional consequences of NPY/NPY1R signaling and found that NPY-mediated suppression of proliferation and promotion of apoptosis was significantly enhanced under conditions of NPY1R overexpression (Figure 4, H and I). Collectively, these results demonstrate that NPY1R activation via NPY signaling opposes the cytoprotective effects of UHRF1 and disrupts epithelial homeostasis.

### The UHRF1/NPY1R axis protects IECs via CREB activation and NF-κB suppression.

**Figure 5:**
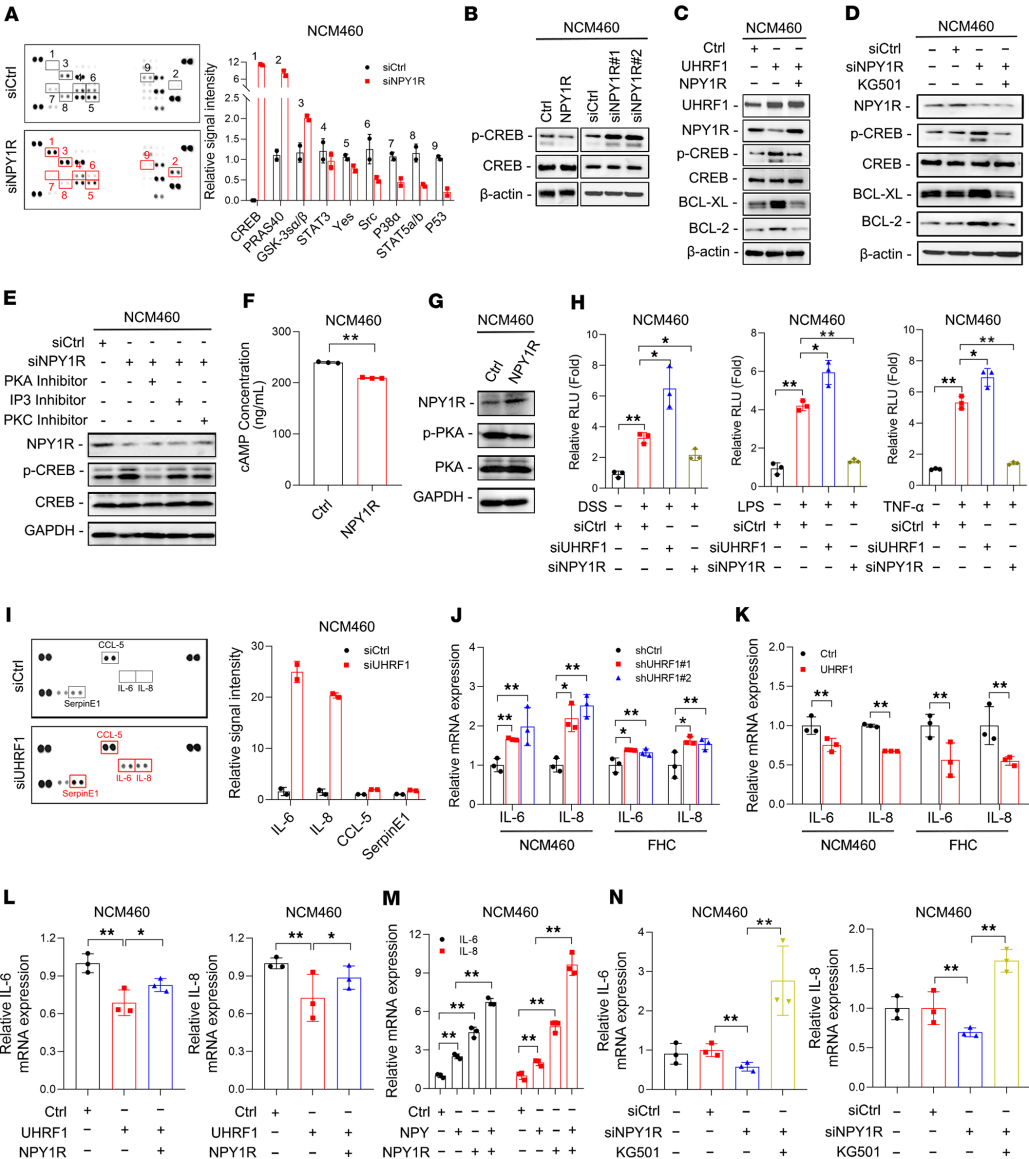
The UHRF1/NPY1R axis protects IECs via CREB activation and NF-κB suppression. (**A**) Immunoblotting and quantification of phosphoproteins in NCM460 cells transfected with siNPY1R or siCtrl. (**B**) Levels of CREB and p-CREB in NCM460 cells after manipulation of NPY1R. (**C**) Levels of the indicated proteins in UHRF1-overexpressing NCM460 cells with or without concurrent NPY1R overexpression. (**D**) Levels of the indicated proteins in NCM460 cells transfected with siNPY1R or siCtrl and treated with or without KG501. (**E**) Levels of NPY1R, CREB, and p-CREB in NCM460 cells transfected with siNPY1R or siCtrl and treated with the indicated inhibitors. (**F**) cAMP concentration in NCM460 cells transfected with NPY1R-overexpressing (NPY1R) or negative control (NC) vector by ELISA. (**G**) Levels of NPY1R, PKA, and p-PKA in NCM460 cells transfected with NPY1R or NC vector. (**H**) Relative luciferase activity in NCM460 cells transfected with siUHRF1, siNPY1R, or siCtrl and treated with or without DSS, LPS, or TNF*-*α. (**I**) Immunoblotting and quantification of cytokines in NCM460 cells transfected with siUHRF1 or siCtrl. (**J**) mRNA expression of IL-6 and IL-8 in NCM460 and FHC cells infected with shUHRF1 or shCtrl. (**K**) mRNA expression of IL-6 and IL-8 in NCM460 and FHC cells infected with UHRF1 or Ctrl. (**L**) mRNA expression of IL-6 and IL-8 in NCM460 cells with or without NPY1R and/or UHRF1 overexpression. (**M**) mRNA expression of IL-6 and IL-8 in NCM460 cells treated with NPY, transfected with NPY1R-overexpressing plasmid, or transfected with the plasmid and treated with NPY. (**N**) mRNA expression of IL-6 and IL-8 measured by qPCR in NCM460 cells transfected with siNPY1R or siCtrl and treated with or without KG501. *P* values were determined by 2-tailed Student’s *t* tests (**F** and **K**) or 1-way ANOVA (**H**, **J**, and **L–N**). Data are shown as mean ± SD. *P < 0.05, **P < 0.01.

To investigate the mechanism by which NPY1R mediates inflammatory damage in IECs, we utilized a human kinase antibody array to identify signaling pathways activated by NPY/NPY1R. Silencing NPY1R in NCM460 cells resulted in substantial changes in the phosphorylation of 9 kinases, most notably a pronounced increase in phosphorylation of cAMP-response element-binding protein (CREB) at Ser133 compared with controls (Figure 5A). Based on the critical function of CREB in controlling cellular responses such as proliferation, survival, and differentiation (24), we sought to determine whether the UHRF1/NPY1R axis regulates IEC viability in a CREB-dependent manner. Western blot analysis showed that NPY1R overexpression reduced CREB phosphorylation (p-CREB), while NPY1R knockdown increased p-CREB levels, with no change in total CREB expression (Figure 5B). Furthermore, UHRF1 overexpression elevated p-CREB and upregulated its downstream antiapoptotic effectors Bcl-2 and Bcl-XL, but these enhancements were almost completely abolished by NPY1R overexpression (Figure 5C). Consistent with these findings, treatment with the CREB inhibitor KG501 abrogated the upregulation of p-CREB, Bcl-2, and Bcl-XL resulting from NPY1R knockdown (Figure 5D). CREB is known to be regulated by upstream pathways including cAMP-PKA, IP_3_-Ca²^+^, and PKC (25–27). To identify which signaling mediator is responsible for NPY/NPY1R-induced CREB activation, we treated NPY1R-knockdown NCM460 cells with specific inhibitors of PKA, IP_3_, or PKC. We found that only PKA inhibition attenuated the increase in p-CREB induced by NPY1R silencing (Figure 5E). Consistent with this, NPY1R overexpression led to significantly reduced cAMP levels and increased PKA phosphorylation (p-PKA) in NCM460 cells (Figure 5, F and G). These results demonstrate that NPY1R suppresses CREB phosphorylation through inhibition of the cAMP/PKA signaling pathway.

The NF-κB pathway, a central mediator of inflammatory signaling, plays a key role in coordinating immune responses (28). Previous research indicates that CREB activation can inhibit NF-κB–dependent transcription by sequestering the shared coactivator CBP (29). We therefore hypothesized that NPY1R activates the NF-κB pathway via suppression of CREB. Consistent with this, UHRF1 knockdown enhanced NF-κB activity, while NPY1R knockdown suppressed it, as measured by NF-κB luciferase reporter assays under LPS, DSS, and TNF-α stimulation (Figure 5H). NF-κB pathway activation is characterized by increased secretion of proinflammatory cytokines. Using a cytokine array, we detected pronounced upregulation of IL-6 and IL-8 in UHRF1-knockdown NCM460 cells (Figure 5I). Notably, as both IL-6 and IL-8 are canonical NF-κB target genes (30), these results further support that the UHRF1/NPY1R axis promotes inflammation through NF-κB activation. UHRF1 knockdown upregulated IL-6 and IL-8 expression in both NCM460 and FHC cells, while UHRF1 overexpression suppressed their production (Figure 5, J and K). Notably, NPY1R overexpression reversed the inhibitory effect of UHRF1 on these cytokines (Figure 5L). Furthermore, combined stimulation with NPY and NPY1R further enhanced IL-6 and IL-8 expression (Figure 5M). Conversely, NPY1R knockdown reduced the secretion of both cytokines, and this suppression was rescued by the CREB inhibitor KG501 (Figure 5N). Collectively, these findings indicate that the UHRF1-mediated NPY1R deficiency confers protection to IECs by promoting CREB activation and inhibiting NF-κB–mediated inflammation.

### miR-141 attenuates NPY1R-induced IEC damage and inflammation.

**Figure 6:**
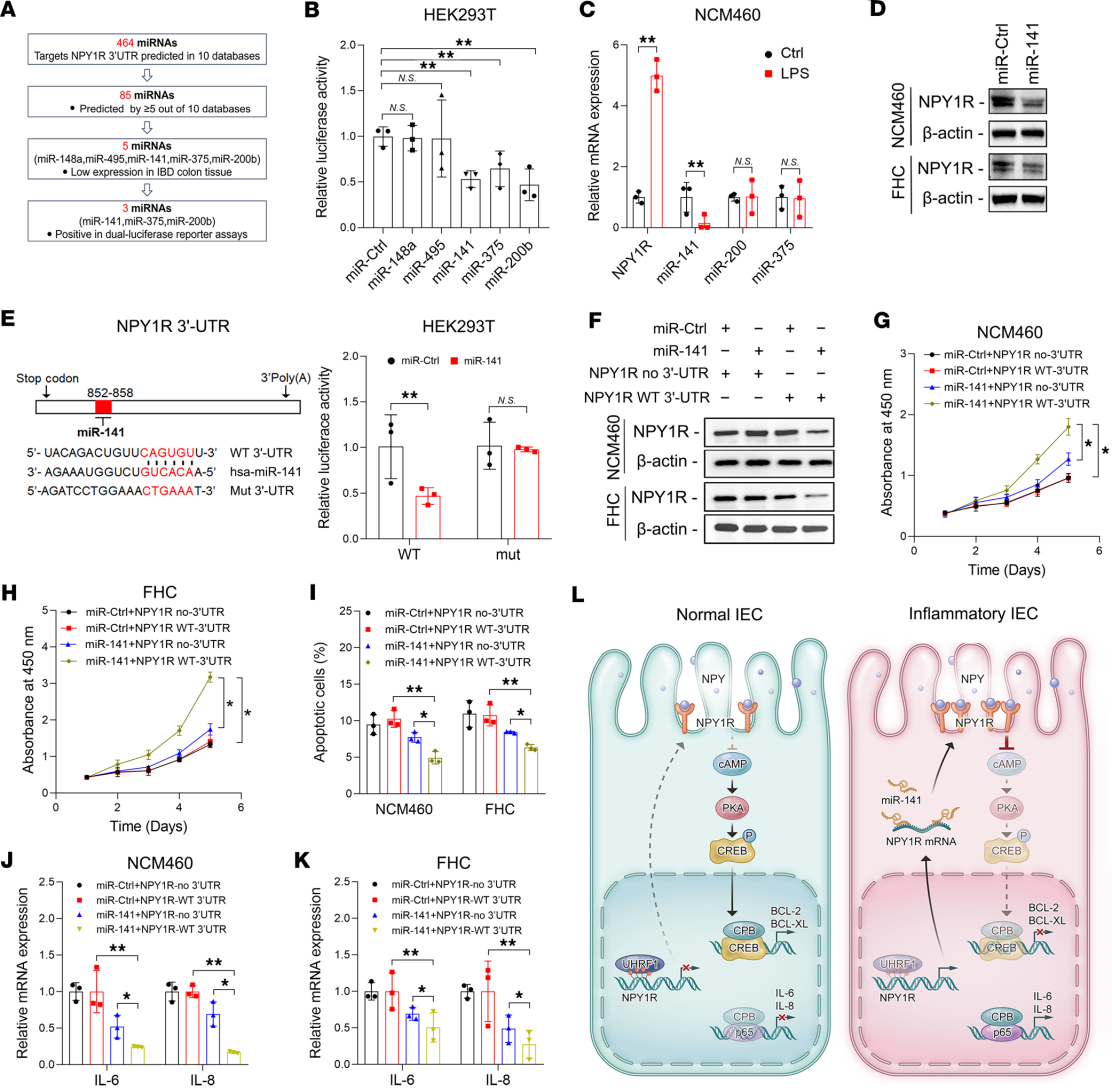
miR-141 attenuates NPY1R-induced IEC damage and inflammation. (**A**) Workflow for the identification of potential miRNAs targeting NPY1R. (**B**) Relative luciferase activity in HEK293T cells cotransfected with the plasmid expressing 3′-UTR of NPY1R and the indicated miRNA mimics or a scramble miRNA control (miR-Ctrl). (**C**) Expression levels of NPY1R and indicated miRNAs in NCM460 cells treated with or without LPS.(**D**) Levels of NPY1R protein in NCM460 and FHC cells transfected with miR-141 mimics (miR-141) or miR-Ctrl. (**E**) Left: Diagram of putative miR-141 binding sites in the 3′-UTR of NPY1R. Right: Relative luciferase activity in HEK293T cells cotransfected with the plasmid expressing WT or mutant (Mut) 3′-UTR of NPY1R and miR-141 or miR-Ctrl. (**F**) Levels of NPY1R protein in NCM460 and FHC cells transfected with NPY1R-overexpressing plasmid with or without WT 3′-UTR and miR-141 or miR-Ctrl. (**G** and **H**) Proliferation of NCM460 (**G**) and FHC (**H**) cells transfected with NPY1R-overexpressing plasmid with or without WT 3′-UTR and miR-141 or miR-Ctrl. (**I**) Apoptotic rates of NCM460 and FHC cells transfected with NPY1R-overexpressing plasmid with or without WT 3′-UTR and miR-141 or miR-Ctrl. (**J** and **K**) mRNA expression of IL-6 and IL-8 in NCM460 (**J**) and FHC (**K**) cells transfected with NPY1R-overexpressing plasmid with or without WT 3′-UTR and miR-141 or miR-Ctrl. (**L**) Proposed working model summarizing this study. *P* values were determined by 1-way ANOVA (**B**, **I**, **J**, and **K**), 2 tailed Student’s *t* tests (**C** and **E**), or 2-way ANOVA (**G** and **H**). Data are shown as mean ± SD. *P < 0.05, **P < 0.01.

microRNAs (miRNAs), which act as negative regulators of gene expression, represent promising therapeutic agents for IBD (31). To identify miRNAs targeting NPY1R, we employed multiple algorithms to predict those with putative binding sites in the 3′-UTR of NPY1R (Figure 6A). From 85 candidate miRNAs (Supplemental Table 2), we selected 5 candidates (miR-148, miR-495, miR-141, miR-375, and miR-200b), which were previously reported to be downregulated in IBD patient samples (32–36), for further validation. Among these, only miR-141, miR-375, and miR-200b significantly inhibited the luciferase activity of an NPY1R 3′-UTR reporter construct (Figure 6B). Notably, LPS stimulation markedly suppressed miR-141 expression in NCM460 cells, whereas miR-375 and miR-200b remained unchanged, suggesting that miR-141 is particularly responsive to inflammatory stimulation and may hold therapeutic potential for reexpression in UC (Figure 6C). Western blot analysis demonstrated that miR-141 overexpression suppressed NPY1R expression in both NCM460 and FHC cells (Figure 6D). Furthermore, dual-luciferase reporter assays confirmed the specificity of this interaction, as mutation of the miR-141 binding sites in the NPY1R 3′-UTR abrogated the suppression of luciferase activity (Figure 6E).

To determine whether miR-141 counteracts NPY1R-induced effects on cell viability and inflammation, we generated NPY1R expression constructs containing or lacking the 3′-UTR (Supplemental Figure 5A). Cotransfection of miR-141 into NCM460 and FHC cells markedly reduced NPY1R expression in the presence of the WT 3′-UTR but not in constructs lacking this region (Figure 6F). Importantly, miR-141 rescued the NPY1R-mediated suppression of proliferation, enhancement of apoptosis, and upregulation of IL-6 and IL-8 (Figure 6, G–K). These effects were markedly attenuated in cells expressing the WT NPY1R construct compared with those expressing the 3′-UTR–deleted variant (Figure 6, G–K). These findings demonstrate that miR-141 represents a promising therapeutic agent for intestinal inflammation by targeting NPY1R (Figure 6L).

## Discussion

Epigenetic modifications, such as DNA methylation, serve as key regulators of gene expression and can modulate IBD pathogenesis by mediating interactions between genetic and environmental factors (37, 38). Studies have shown that the loss of DNA methylation in the IFNG promoter is associated with more severe disease progression in patients with IBD (39). Patients with IBD showed a lower degree of methylation of the STAT4 promoter than did the healthy controls (40). Additionally, methylation of TGF-β and IL-6 can serve as alternative biomarkers for diagnosing pediatric IBD (41). Based on these findings, investigating DNA methylation may, therefore, reveal potential molecular targets for the diagnosis and treatment of IBD. In this study, we investigated the role of UHRF1 in driving IEC damage during IBD. UHRF1 contains multiple functional domains that interact with various chromatin-modifying proteins to regulate DNA methylation, histone modifications, cell proliferation, and DNA repair, thereby exerting diverse biological functions (42). Previous studies have shown that loss of UHRF1 increases cellular sensitivity to DNA damage (43); subsequent research further demonstrates that UHRF1 can recognize interstrand crosslink damage and interact with DNA repair nucleases, suggesting its potential role as a promoter of DNA damage repair (44). Beyond the established role of UHRF1 in development, our study reveals that its heterozygous deletion is sufficient to trigger an IBD-like phenotype in mice, characterized by shortened colon length, tissue architecture disruption, and elevated apoptosis of IECs. These results propose UHRF1 haploinsufficiency as a potential predisposing factor for IBD, suggesting that genetic or epigenetic reductions in UHRF1 levels could underlie susceptibility in a subset of patients. Mechanistically, we demonstrate that the protective effect of UHRF1 on IECs is dependent on its regulation of NPY1R methylation. Early studies on NPY1R have primarily focused on the nervous system. Research in depression models has shown that changes in NPY1R levels can alleviate depressive-like behaviors and enhance brain-derived neurotrophic factor (BDNF) expression, highlighting its critical role in emotional regulation and neurogenesis (45). Further studies have demonstrated that NPY1R-expressing neurons in the lateral septum act as a hub integrating homeostatic and hedonic control circuits, coordinating feeding and emotional behaviors and thereby mitigating stress responses (46). More recent evidence indicates that NPY1R may also be involved in the development and progression of various diseases, including cancer, diabetes, and pain (47, 48). Our study further reveals that, in IECs, UHRF1 directly binds to the NPY1R promoter, inducing hypermethylation and suppressing its expression, thus uncovering a potentially novel regulatory mechanism and biological significance of NPY1R outside the nervous system. The mechanism underlying UHRF1 downregulation in IECs of patients with IBD remains unclear but likely involves multifaceted stress responses from a disturbed microenvironment. Proinflammatory cytokines such as TNF-α and IL-1β can suppress UHRF1 transcription via NF-κB and p38 MAPK pathways (14). Chronic inflammation also promotes epigenetic dysregulation, including aberrant DNA methylation at the UHRF1 locus (49), while dysregulated miRNAs may also contribute to UHRF1 silencing through mRNA degradation (50).

IECs play a crucial role in the onset and progression of IBD. Although immune cells remain the primary source of inflammatory mediators, emerging evidence suggests that IEC are not merely a passive barrier but also active contributors to inflammation. Activated IEC can secrete various cytokines and chemokines, actively participating in and amplifying the inflammatory response (51, 52). In patients with IBD, IEC function is compromised, resulting in disruption of the intestinal barrier, increased intestinal permeability, and subsequent infiltration of exogenous pathogens and endogenous immune activators into the intestinal wall (53). This triggers sustained immune responses and chronic inflammation (54). Our study revealed that UHRF1 knockdown or NPY1R overexpression aggravated IEC damage in IBD mice. Moreover, UHRF1 knockdown markedly enhanced NF-κB luciferase reporter activity in response to inflammatory stimuli, whereas NPY1R knockdown exerted the opposite effect. These findings highlight the active immunomodulatory role of IECs in intestinal inflammation. These mechanisms align precisely with our observation that UHRF1 deficiency triggers abnormal IL-6 and IL-8 upregulation. Our results indicate that the protective effect of UHRF1 on IECs, mediated through inhibition of NPY1R methylation, is associated with the transcription factor CREB. Previous studies have reported that CREB overexpression inhibits NF-κB–mediated transcription by competing for the limited coactivator CBP (29). Furthermore, we demonstrated that the UHRF1/NPY1R axis protects IECs by activating CREB and suppressing NF-κB, and it reduces the secretion of inflammatory factors, thereby alleviating IBD pathology. In addition, previous studies have shown that the loss of UHRF1 and DNMT1 can induce an immune response in zebrafish livers by triggering viral mimicry through transposable elements (55), highlighting the need to evaluate transposon activity and innate immunity in IBD models to further investigate this potential connection.

miRNAs functioning as negative regulators of gene expression have emerged as promising therapeutic candidates for IBD (56). miRNAs are a class of noncoding RNA molecules that play a crucial role in mRNA translation suppression or degradation by binding to complementary target mRNA (57). Several studies have confirmed the importance of miRNAs in regulating gut barrier function and inflammation signaling, suggesting their potential in the treatment of IBD. For instance, miR-31 alleviates inflammation in mouse colonic epithelium by suppressing the expression of IL-7R, IL-17RA, and GP130 (58). Similarly, synthetic miR-223 mimics administered in lipid nanoparticle emulsions successfully reduced occult bleeding, weight loss, and edema in DSS-induced colitis in mice (59). In this study, we highlight miR-141 as a potential candidate that may rescue IEC damage by inhibiting NPY1R expression. The colonic administration of the miR-141 precursor has been shown to alleviate colitis in TNBS-induced and IL-10–KO mice (60). Thus, exogenous administration of miR-141 represents a promising therapeutic strategy for treating IBD by targeting NPY1R.

In conclusion, our study elucidates the UHRF1/NPY1R axis as a critical epigenetic mechanism in intestinal inflammation and identifies miR-141 as a negative regulator of NPY1R, highlighting its potential as a therapeutic agent. Our findings not only reveal the pivotal role of UHRF1 in maintaining IEC homeostasis via epigenetic regulation but also provide potentially novel molecular targets and theoretical insights for miRNA-based IBD interventions.

## Methods

### Sex as a biological variable.

Both male and female mice were included in the experimental study, and both male and female patients were included in the clinical cohort. Sex was not considered as a biological variable in the statistical analysis for this study.

### Patient recruitment and sample collection.

Twenty-five patients with UC and 25 controls undergoing routine endoscopy were recruited from Xijing Hospital of Digestive Diseases (Xi’an, China). Written informed consent was obtained from all participants prior to enrollment. Demographic characteristics, including age, sex, disease severity, and treatment information, are provided in Supplemental Table 1.

### Murine colitis models.

To induce acute colitis, sex- and age-matched mice (6–8 weeks old) received drinking water containing 2.5% (wt/vol) DSS for 5 days, followed by normal water for 2 days. Body weight, stool consistency, and rectal bleeding were monitored daily, and the DAI was recorded. The AIEC strain was provided by Arlette Darfeuille-Michaud (Clermont Auvergne University, France). Bacteria were cultured aerobically at 37°C in Luria-Bertani (LB) medium supplemented with antibiotics overnight with agitation. Mice housed under standard specific pathogen–free (SPF) conditions were orally gavaged with 1 × 10^9^ CFU of AIEC per mouse daily for 14 consecutive days. At the end of the treatment, mice were sacrificed for downstream analyses.

### Cell culture.

The human IEC lines NCM460 and FHC were prepared by Department of Immunology (The Fourth Military Medical University, China). Human FHC and NCM460 were maintained in Dulbecco’s modified Eagle’s medium (DMEM) supplemented with 10% FBS, 100 U/mL penicillin, and 100 μg/mL streptomycin at 37°C in a humidified atmosphere containing 5% CO_2_. All cell lines were authenticated by short tandem repeat (STR) profiling, and routine testing confirmed the absence of mycoplasma contamination.

### Constructs, oligonucleotides, infection, and transfection.

Lentiviral vectors encoding short hairpin RNA (shRNA) sequences with EGFP-puromycin-FLAG were generated using the GV493 backbone (GeneChem, Shanghai, China) and designated as shUHRF1, shNPY1R, or shCtrl (a nontargeting shRNA control). Lentiviral vectors carrying human UHRF1 or NPY1R cDNAs with EGFP-puromycin-FLAG were also constructed by GeneChem. Chemically modified small interfering RNAs (siRNAs) targeting UHRF1, DNMT1, or NPY1R were purchased from GenePharma (Shanghai, China) for gene knockdown. Expression vectors for UHRF1 and NPY1R were generated using the pcDNA3.1 and pEX-3 backbones, respectively (GeneChem). FHC and NCM460 cells were infected with lentiviruses in the presence of 5 μg/mL polybrene and selected with 2 μg/mL puromycin for 2 weeks. Transfection of siRNAs or plasmids was performed using jetPRIME Transfection Reagent (Polyplus Transfection, Illkirch, France) according to the manufacturer’s instructions.

### IHC.

IHC staining for the target molecules was performed on single sections from clinical FFPE tissues and colitis mouse samples. The samples were deparaffinized and subjected to heat-mediated antigen retrieval using Tris/EDTA buffer (pH 9.0; for UHRF1) or 10 mM sodium citrate buffer (pH 6.0; for NPY1R), according to the manufacturer’s instructions. The slides were then treated with 3% H_2_O_2_ and incubated overnight at 4°C with primary antibodies. The primary antibody information is listed in Supplemental Table 3. Subsequently, the slides were incubated with secondary antibodies, and the signal was visualized using DAB^+^. Nuclei were counterstained with hematoxylin. Staining intensity was assessed as previously described (61).

### Protein extraction and Western blot.

Cultured cells and tissue samples were lysed in RIPA buffer (Beyotime) supplemented with a protease inhibitor cocktail and a phosphatase inhibitor (Roche). Protein extracts were separated by SDS-PAGE and transferred onto nitrocellulose membranes (Millipore), which were blocked with 10% nonfat milk and then incubated sequentially with primary antibodies followed by HRP-conjugated secondary antibodies (Cell Signaling Technology). Detailed information on primary antibodies is listed in Supplemental Table 3. Protein bands were visualized using enhanced chemiluminescence reagents (ZETA) and imaged with a Molecular Imager ChemiDoc XRS+ System using Image Lab software (Bio-Rad).

### RNA extraction and qPCR.

Total RNA was extracted from cultured cells and fresh-frozen mucosal tissues using the miRNeasy kit (QIAGEN) according to the manufacturer’s instructions. RNA quality was assessed by measuring the A260/A280 absorbance ratio. Complementary DNA (cDNA) for miRNA detection was synthesized using a TaqMan miRNA reverse transcription kit (Takara). For mRNA detection, 500 ng of total RNA was reverse transcribed into cDNA in a 10 μL reaction using the PrimeScript RT Master Mix kit (Takara). Quantitative PCR (qPCR) was performed in triplicate using SYBR Premix Ex Taq II (Takara) on a CFX96 Real-Time PCR Detection System (Bio-Rad). All primers were designed and synthesized by Takara. U6 and GAPDH served as internal controls. The sequences of primers used for qPCR are listed in Supplemental Table 4.

### Cell proliferation assay.

Cells were seeded in 96-well plates at a density of 2.5 × 10^3^ cells/well. After a 24-h incubation, cells were treated with medium, LPS, TNF-α, or 2% DSS and incubated at 37°C in 5% CO_2_ for a total of 5 days. Cell viability was assessed daily by incubation with CCK-8 solution. Absorbance at 450 nm was measured using a Varioskan Flash spectrometer (Thermo Fisher Scientific).

### Cell apoptosis assay.

Apoptosis of cultured cells was assessed by flow cytometry using either the FITC Annexin V Apoptosis Detection Kit (BD Pharmingen) or the Annexin V-PE/7-AAD Apoptosis Kit (BD Biosciences). Briefly, cells were seeded in 24-well flat-bottom plates and treated with LPS, TNF-α, or 2% DSS for 24 h. Cells were then harvested, washed with PBS, fixed, and stained according to the manufacturer’s instructions. The proportion of apoptotic cells that included both early and late apoptotic populations was determined.

### Generation of UHRF1 conditional knockout mice.

Floxed mice (*Uhrf1*^fl/fl^), with LoxP sites flanking exon 4 of *Uhrf1*, were purchased from Shanghai Model Organisms Center, Inc. (NM-CKO-00094). Villin-Cre transgenic mice, expressing Cre recombinase specifically in IECs, were obtained from Jackson Laboratory. WT C57BL/6J mice were purchased from C57BL/6J mice (4-week-old) were obtained from the Experimental Animal Center of Fourth Military Medical University. Female *Uhrf1*^fl/fl^ mice were crossed with male Villin-Cre transgenic mice, where Cre is expressed under the control of the *villin* gene regulatory region, generating intestinal epithelium-specific UHRF1-KO mice (*Uhrf1*^fl/fl^ Villin-Cre). The resultant *Uhrf1*^fl/fl^ mice expressing Cre were designated as *Uhrf1*^IEC−/−^ mice, heterozygous mice (*Uhrf1*^IEC[+/−]^) exhibiting partial deletion were included as an intermediate group, and cohoused littermates lacking Cre (*Uhrf1*^IEC[+/+]^) were used as WT controls. All mice were on a C57BL/6 background and housed under SPF conditions in the animal facility of the Fourth Military Medical University. Age-matched littermates were used for all experiments. The primers for identification of genotype are listed in Supplemental Table 4. Histological score was assessed as previously described (62).

### DNA microarray analysis.

Total RNA was extracted from NCM460-shCtrl and NCM460-shUHRF1 cells using Trizol reagent (Invitrogen) and purified with the mirVana miRNA Isolation Kit (Ambion) according to the manufacturer’s instructions. Complementary DNA (cDNA) labeled with the fluorescent dye Cy3-dCTP was generated using Eberwine’s linear RNA amplification method followed by enzymatic reactions. The fragmented cmDNA was hybridized onto Agilent Human (V2) Gene Expression 8 × 60K microarrays (Agilent Technologies). Data normalization and identification of differentially expressed genes were performed using Greenspring software V13 (Agilent Technologies).

### DNA methylation array analysis.

Genomic DNA was isolated from NCM460-shCtrl and NCM460-shUHRF1 cells using the QIAamp DNA Mini Kit (Qiagen). DNA purity and concentration were measured with a Nanodrop 2000 spectrophotometer (Thermo Scientific). Approximately 500 ng of DNA from each sample was subjected to sodium bisulfite conversion using the EZ DNA methylation–Gold Kit (Zymo Research) according to the manufacturer’s protocol. Genome-wide DNA methylation was assessed using the Illumina Infinium HumanMethylation850K BeadChip (Illumina) following the manufacturer’s instructions. Array data (IDAT files) were analyzed with the ChAMP package in R to determine methylation levels. The methylation status of each probe was represented as a β value, defined as the ratio of methylated probe intensity to total probe intensity (sum of methylated and unmethylated intensities plus a constant α, α = 100). CpG sites with |Δβ| ≥ 0.20 (test vs. control) and an adjusted *P* ≤ 0.05 were considered differentially methylated. A CpG site was classified as hypermethylated if Δβ ≥ 0.20 or hypomethylated if Δβ ≤ −0.20.

### Methylated DNA bisulfite sequencing.

Genomic DNA was extracted and bisulfite-converted using the EpiTect Bisulfite Kit (Qiagen) according to the manufacturer’s instructions. The amplified PCR products were cloned into the T-Vector pMD19 (Takara), and 10 clones per sample were sequenced. The primers used are listed in Supplemental Table 4.

### Human antibody array.

Human phospho-kinase arrays were obtained from R&D Systems. According to the manufacturer’s protocol, cells were seeded at 1 × 10^7^ cells/mL in 6-well plates and cultured to 80% confluence. Cells were then lysed in the provided lysis buffer, and 300 μg of cellular protein lysates were incubated with the microarray. The phosphorylation levels of 37 commonly studied kinases were quantified using the WesternBright ECL kit (InCellGene). A human cytokine antibody array was used to simultaneously detect and quantify 36 cytokines in NCM460 cells treated with siNPY1R or siCtrl, following the manufacturer’s instructions.

### ChIP.

ChIP assays were performed using the Pierce Agarose ChIP Kit (Thermo Fisher Scientific), and ChIP-enriched DNA samples were analyzed by PCR. Briefly, chromatin-protein complexes were incubated with UHRF1 antibody or rabbit IgG and then immunoprecipitated using Protein A/G PLUS Agarose (Thermo Fisher Scientific). The precipitated immune complexes were washed, and DNA was extracted. The purified DNA was subjected to PCR to amplify the binding sites within the NPY1R promoter region, and PCR products were visualized by gel electrophoresis. For quantitative analysis, qPCR was performed using SYBR Premix Ex Taq II (Takara) on a CFX96 Real-Time PCR Detection System (Bio-Rad) to quantify the enrichment of target sequences, with results normalized to input DNA. The primers used for qPCR are listed in Supplemental Table 4.

### ELISA.

cAMP concentration in the cell supernatants was quantified using an ELISA kit (Cloud-Clone, Wuhan, China). Briefly, samples were loaded into 96-well plates and analyzed in triplicate. Detection antibodies and enzyme conjugates were then sequentially added, followed by washing with the kit-provided wash buffers, according to the manufacturer’s protocol. Absorbance was measured at 450 nm using a Thermo Fisher Scientific Varioskan Flash multimode plate reader.

### Luciferase reporter assay.

For 3′-UTR luciferase reporter assays, the WT and mutant constructs of the 3′-UTR of NPY1R were subcloned into a psiCHECK-2 vector. Cells were cotransfected with psiCHECK-2-NPY1R 3′-UTR WT or mutant plasmids and miR-141 mimics or a negative control. The firefly and Renilla luciferase activities were measured using the Dual-Luciferase Reporter Assay System (Promega). Firefly luciferase activity was normalized to Renilla activity and is presented as relative luciferase activity.

### Statistics.

Statistical analyses were performed using SPSS 27.0 (version 4.4.1). Each experiment was conducted in triplicate or more. Data are presented as mean ± SD. Statistical significance was defined as *P* < 0.05. Depending on the type of experiment, comparisons were made using 2-tailed Student’s *t* tests, 1-way or 2-way ANOVA, or χ^2^ tests.

### Study approval.

The study was approved by the Institutional Research Medical Ethics Committee of Xijing Hospital (no. NFEC-2014-014) and conducted in accordance with the Declaration of Helsinki. All mouse experiments were approved by the Experimental Animal Ethics Committee of the Fourth Military Medical University (no. IACUC-20190923) and conducted in accordance with institutional regulations.

### Data availability.

All data needed to evaluate the conclusions in the paper are present in the paper and/or the supplemental materials. Additional data related to this paper may be requested from the corresponding author. Values for all data points in graphs are reported in the Supporting Data Values file. The complete DNA microarray and DNA methylation data have been deposited in the Gene Expression Omnibus (GEO) under accession nos. GSE310603 (DNA microarray) and GSE310605 (DNA methylation). These data are publicly accessible.

## Author contributions

YH, Y Liu, X Zhang, HL, and HZ performed the experiments. YH, LS, Y Liu, and X Zhang collected, analyzed and interpreted the data. XR, YF, FW, HF, DF, and Y Lu supervised the experiments. JC and DL accessed and verified the data. YH, LS, and XB wrote the manuscript. X Zhao revised the manuscript. XB, YF, KW, and X Zhao were responsible for the decision to submit the manuscript. X Zhao supervised the study and designed the experiments. All authors read and approved the final version of the manuscript.

## Funding support

- National Natural Science Foundation of China (82341223, 82573226, and 82425046)
- Natural Science Basic Research Program of Shaanxi Province (2023-JC-QN-0829, 2025JC-YBMS-972).

## Supplementary Material

Supplemental data

Unedited blot and gel images

Supporting data values

## Version 1 02/09/2026

Electronic publication
