# Supplementary material for: UHRF1 deficiency exacerbates intestinal inflammation by epigenetic modulation of NPY1R gene methylation
Source: JCI Insight. 2026 Feb 9;11(3):e190894. doi: 10.1172/jci.insight.190894 (PMC12892884; doi:10.1172/jci.insight.190894)
Supplement: Supplemental data [file jciinsight-11-190894-s018.pdf]

Figure S1

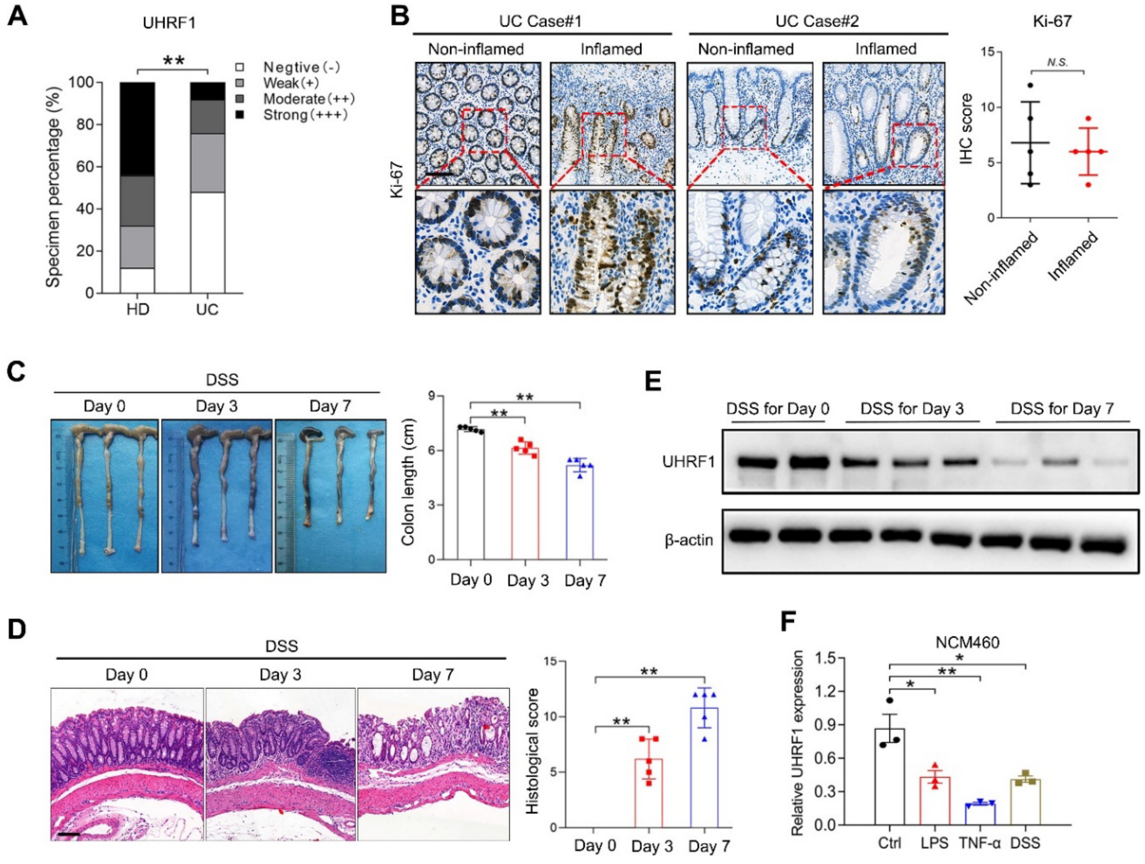

Figure S1. UHRF1 is downregulated in IECs under inflammatory conditions.

(A) Quantification and positive staining percentage of UHRF1 in colonic tissues from healthy donors and UC patients. **(B)** Representative staining images and quantification of Ki-67 in the indicated colonic tissues from non-inflamed and inflamed tissues from UC patients. Scale bar: 50 μm. **(C)** Representative image and quantification of colon length from mice treated with DSS and sacrificed at different time points (n=5). **(D)** Representative H&E staining and histological score of colon tissues from mice treated with DSS and sacrificed at different time points (n=5). Scale bar:100 μm. **(E)** Levels of UHRF1 protein in colon tissues from mice treated with DSS and sacrificed at different time points. **(F)** mRNA expression of UHRF1 in NCM460 cells serum-starved for 12 h prior to treatment with LPS, TNF-α, or DSS. *P*-values were determined by Chi square test (A), 2-tailed Student's t tests (B), or One-way ANOVA (C, D, F). Data are mean ± SD. *$P < 0.05$, **$P < 0.01$. *N.S.*, not significant.

Figure S2

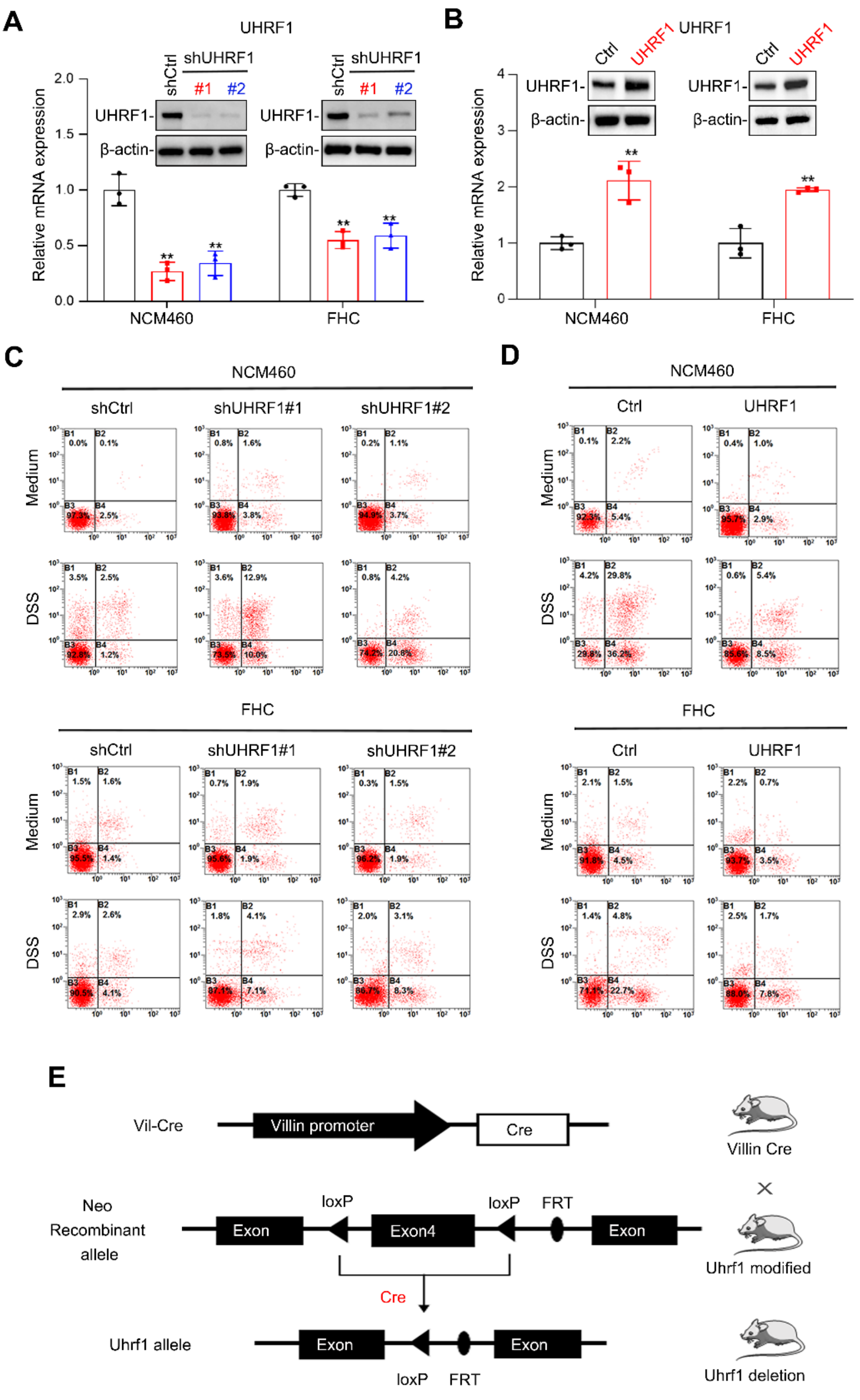

Figure S2. UHRF1 deficiency sensitize IECs to inflammatory damage.

(A) mRNA and protein expression of UHRF1 in NCM460 and FHC cells infected with shUHRF1 or shCtrl. **(B)** mRNA and protein expression of UHRF1 in NCM460 and FHC cells infected with UHRF1 or Ctrl. **(C)** Flow

cytometric analysis and representative plots of NCM460 and FHC infected with shUHRF1 or shCtrl. **(D)** Flow cytometric analysis and representative plots of NCM460 and FHC infected with UHRF1 or Ctrl. **(E)** Schematic representation of the generation of the *Uhrf1* conditional knockout allele using the Villin-Cre system in mice. P-values were determined by One-way ANOVA (A) or 2-tailed Student's t tests (B). Data are mean ± SD. $^{**}P < 0.01$.

Figure S3

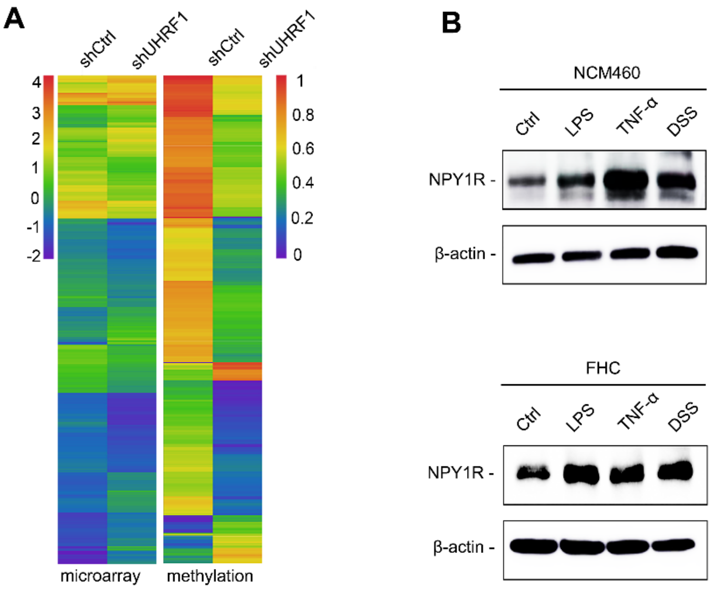

Figure S3. UHRF1 represses NPY1R expression through promoter methylation in IECs.

(A) Heatmaps showing mRNA expression and DNA methylation changes of genes in NCM460 cells transfected with shUHRF1 or shCtrl. **(B)** Levels of NPY1R protein in NCM460 and FHC cells treated with LPS, TNF-α, or DSS.

Figure S4

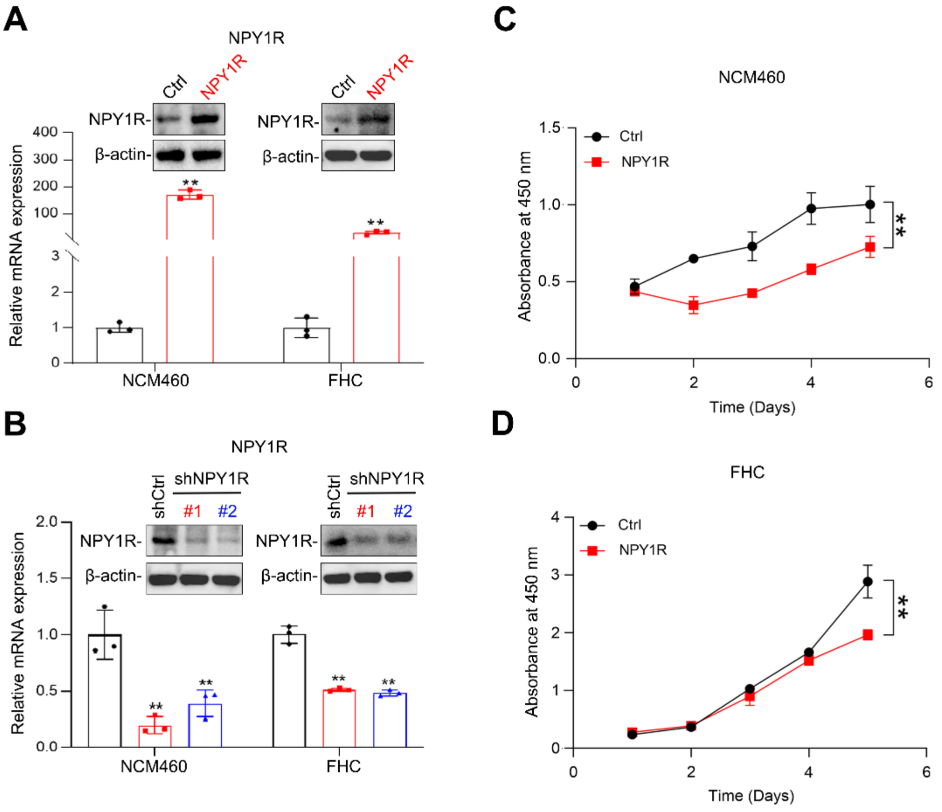

Figure S4. NPY1R activation antagonizes UHRF1-mediated protection in IECs.

(A) mRNA and protein expression of NPY1R in NCM460 and FHC cells infected with NPY1R or Ctrl. **(B)** mRNA and protein expression of NPY1R in NCM460 and FHC cells infected with shNPY1R or shCtrl. **(C and D)** Proliferation of NCM460 (C) and FHC (D) cells infected with NPY1R and Ctrl. *P*-values were determined by 2-tailed Student's t tests (A), One-way ANOVA (B), or Two-way ANOVA (C, D). Data are mean ± SD. $^{**}P < 0.01$.

Figure S5

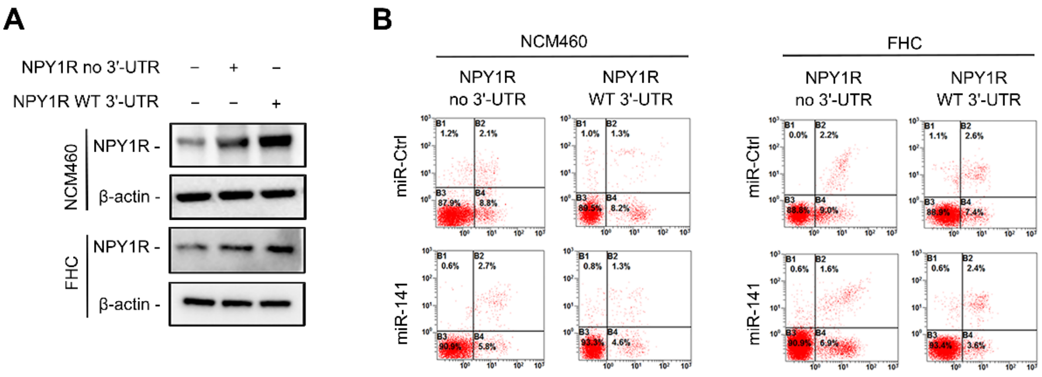

Figure S5. miR-141 attenuates NPY1R-induced IEC damage and inflammation.

(A) mRNA and protein expression of NPY1R in NCM460 and FHC cells transfected with NPY1R plasmid with or without wild-type (WT) 3'-UTR. **(B)** Flow cytometric analysis and representative plots of NCM460 and FHC cells transfected with NPY1R-overexpressing plasmid with or without WT 3'-UTR and miR-141 or miR-Ctrl.

Supplementary Table 1. The characteristics of patients with ulcerative colitis.

No.	Gender	Age (years)	Disease duration (months)	Extent	Severity
1	Male	20	15	E2	S3
2	Male	35	10	E2	S3
3	Male	47	46	E2	S1
4	Female	55	3	E2	S1
5	Male	65	12	E2	S2
6	Female	61	6	E2	S1
7	Male	47	18	E2	S2
8	Male	53	9	E2	S2
9	Female	26	24	E2	S1
10	Male	30	33	E2	S2
11	Male	43	60	E2	S2
12	Male	38	5	E2	S2
13	Male	49	36	E2	S2
14	Male	68	23	E2	S1
15	Male	22	16	E2	S3
16	Male	18	9	E3	S3
17	Female	55	45	E3	S1
18	Female	39	49	E1	S1
19	Female	47	53	E3	S1
20	Female	46	63	E3	S1
21	Female	56	36	E3	S2
22	Female	40	48	E1	S2
23	Male	28	15	E3	S3
24	Female	35	24	E3	S1
25	Male	60	26	E3	S2

[a] E1, proctitis; E2, left-sided colitis; E3, extensive colitis.

[b] S0, remission; S1, mild UC; S2, moderate UC; S3, severe UC.

Supplementary Table 2. miRNAs targeting NPY1R predicted by multiple algorithms.

No.	miRNA	Gene symbol	StemLoop ID
1	hsa-miR-515-5p	NPY1R	hsa-mir-515-2
2	hsa-miR-622	NPY1R	hsa-mir-622
3	hsa-miR-148b	NPY1R	hsa-mir-148b
4	hsa-miR-148a	NPY1R	hsa-mir-148a
5	hsa-miR-223	NPY1R	hsa-mir-223
6	hsa-miR-144	NPY1R	hsa-mir-144
7	hsa-miR-338-5p	NPY1R	hsa-mir-338
8	hsa-miR-548d-3p	NPY1R	hsa-mir-548d-2
9	hsa-miR-495	NPY1R	hsa-mir-495
10	hsa-miR-152	NPY1R	hsa-mir-152
11	hsa-miR-567	NPY1R	hsa-mir-567
12	hsa-miR-607	NPY1R	hsa-mir-607
13	hsa-miR-568	NPY1R	hsa-mir-568
14	hsa-miR-380	NPY1R	hsa-mir-380
15	hsa-miR-633	NPY1R	hsa-mir-633
16	hsa-miR-656	NPY1R	hsa-mir-656
17	hsa-miR-381	NPY1R	hsa-mir-381
18	hsa-miR-636	NPY1R	hsa-mir-636
19	hsa-miR-549	NPY1R	hsa-mir-549
20	hsa-miR-300	NPY1R	hsa-mir-300
21	hsa-miR-218	NPY1R	hsa-mir-218-2
22	hsa-miR-548c-3p	NPY1R	hsa-mir-548c
23	hsa-miR-526b	NPY1R	hsa-mir-526b
24	hsa-miR-141	NPY1R	hsa-mir-141
25	hsa-miR-573	NPY1R	hsa-mir-573
26	hsa-miR-101	NPY1R	hsa-mir-101-2
27	hsa-miR-548a-5p	NPY1R	hsa-mir-548a-3
28	hsa-miR-371-5p	NPY1R	hsa-mir-371
29	hsa-miR-433	NPY1R	hsa-mir-433
30	hsa-miR-374b	NPY1R	hsa-mir-374b
31	hsa-miR-524-5p	NPY1R	hsa-mir-524
32	hsa-miR-142-5p	NPY1R	hsa-mir-142
33	hsa-miR-548a-3p	NPY1R	hsa-mir-548a-3
34	hsa-miR-374a	NPY1R	hsa-mir-374a
35	hsa-miR-331-5p	NPY1R	hsa-mir-331
36	hsa-miR-511	NPY1R	hsa-mir-511-2
37	hsa-miR-548d-5p	NPY1R	hsa-mir-548d-2
38	hsa-miR-520d-5p	NPY1R	hsa-mir-520d
39	hsa-miR-558	NPY1R	hsa-mir-558
40	hsa-miR-579	NPY1R	hsa-mir-579
41	hsa-miR-603	NPY1R	hsa-mir-603
42	hsa-miR-375	NPY1R	hsa-mir-375

43	hsa-miR-1301	NPY1R	hsa-mir-1301
44	hsa-miR-216b	NPY1R	hsa-mir-216b
45	hsa-miR-224	NPY1R	hsa-mir-224
46	hsa-miR-522	NPY1R	hsa-mir-522
47	hsa-miR-559	NPY1R	hsa-mir-559
48	hsa-miR-200c	NPY1R	hsa-mir-200c
49	hsa-miR-582-5p	NPY1R	hsa-mir-582
50	hsa-miR-605	NPY1R	hsa-mir-605
51	hsa-miR-377	NPY1R	hsa-mir-377
52	hsa-miR-425	NPY1R	hsa-mir-425
53	hsa-miR-453	NPY1R	hsa-mir-453
54	hsa-miR-654-3p	NPY1R	hsa-mir-654
55	hsa-miR-208b	NPY1R	hsa-mir-208b
56	hsa-miR-200b	NPY1R	hsa-mir-200b
57	hsa-miR-194	NPY1R	hsa-mir-194-2
58	hsa-miR-18b	NPY1R	hsa-mir-18b
59	hsa-miR-410	NPY1R	hsa-mir-410
60	hsa-miR-655	NPY1R	hsa-mir-655
61	hsa-miR-920	NPY1R	hsa-mir-920
62	hsa-miR-212	NPY1R	hsa-mir-212
63	hsa-miR-130a	NPY1R	hsa-mir-130a
64	hsa-miR-513a-3p	NPY1R	hsa-mir-513a-2
65	hsa-miR-153	NPY1R	hsa-mir-153-2
66	hsa-miR-106b	NPY1R	hsa-mir-106b
67	hsa-miR-586	NPY1R	hsa-mir-586
68	hsa-miR-18a	NPY1R	hsa-mir-18a
69	hsa-miR-105	NPY1R	hsa-mir-105-2
70	hsa-miR-488	NPY1R	hsa-mir-488
71	hsa-miR-1283	NPY1R	hsa-mir-1283-2
72	hsa-miR-132	NPY1R	hsa-mir-132
73	hsa-miR-507	NPY1R	hsa-mir-507
74	hsa-miR-548b-5p	NPY1R	hsa-mir-548b
75	hsa-miR-548c-5p	NPY1R	hsa-mir-548c
76	hsa-miR-20a	NPY1R	hsa-mir-20a
77	hsa-miR-429	NPY1R	hsa-mir-429
78	hsa-miR-664	NPY1R	hsa-mir-664
79	hsa-miR-1249	NPY1R	hsa-mir-1249
80	hsa-miR-532-5p	NPY1R	hsa-mir-532
81	hsa-miR-200a	NPY1R	hsa-mir-200a
82	hsa-miR-369-3p	NPY1R	hsa-mir-369
83	hsa-miR-431	NPY1R	hsa-mir-431
84	hsa-miR-657	NPY1R	hsa-mir-657
85	hsa-miR-450b-5p	NPY1R	hsa-mir-450b

Supplementary Table 3. Antibodies and reagents used in this study.

Reagents or Antibodies	Source	Identifier
Anti-UHRF1 (H-8)	Santa Cruz Biotechnology	sc-393192
Anti-UHRF1 [EPR18803]	Abcam	ab194236
Anti-β-actin	Cell Signaling Technology	#2281
Anti-NPY1R (E-4)	Santa Cruz Biotechnology	sc-393192
Anti-DNMT1 (D63A6)	Cell Signaling Technology	#5032
Anti-DNMT1	NOVUS	NB100-56519
Anti-CREB	Cell Signaling Technology	#48H2
Anti-p-CREB (ser133)	Cell Signaling Technology	#87G3
Anti-mouse IgG, HRP-linked Antibody	Cell Signaling Technology	#7076
Anti-rabbit IgG, HRP-linked Antibody	Cell Signaling Technology	#7074
DSS	AppliChem Panreac	A3261,0250
CREB-inhibitor (KG501)	Selleck	S8409
Human TNF-α	Pepro Tech	300-01A
LPS	Solarbio	L8880

Supplementary Table 4. Sequences of primers used in this study.

Primer	Sequence
UHRF1 (human)	F: GACAAGCAGCTCATGTGCGATG<br>R: AGTACCACCTCGCTGGCATCAT
IL-6 (human)	F: TACCCCCAGGAGAAGATTCC<br>R: TTTTCTGCCAGTGCCTCTTT
IL-8 (human)	F: GAGAGTGATTGAGAGTGGACCAC<br>R: CACAACCCTCTGCACCCAGTTT
GAPDH (human)	F: GACAGTCAGCCGCATCTTCT<br>R: GCGCCCAATACGACCAAATC
NPY1R (human)	F: CCATCGGACTCTCATAGGTTGTC<br>R: GACCTGTACTTATTGTCTCTCATC
UHRF1(mouse)	F: AGACCTCTCTGGCAACAAGCGT<br>R: AGTCTTCAGCCTCCGCACCTTT
NPY1R(mouse)	F: CCATCTGACTCTCACAGGCTGT<br>R: TCTTGTCCATCATGTTGTTTCTCC
GAPDH (mouse)	F: CATCACTGCCACCCAGAAGACTG<br>R: ATGCCAGTGAGCTTCCCGTTCAG
Villin-Cre (wild-type)	F: GCCTTCTCCTCTAGGCTCGT<br>R: TATAGGGCAGAGCTGGAGGA
Villin-Cre (knockout)	F: GCCTTCTCCTCTAGGCTCGT<br>R: AGGCAAATTTTGGTGTACGG
Uhrf1$^{fl/fl}$	F: TTGCTGCCAGGTAGGACA<br>R: GAAGAGGCGGCTCAGGAGA
NPY1R-BSP-1	F: TGATTGTTTTGATTTTTTAAAGG<br>R: AAAAAAAAACAATCCAACAAAA
NPY1R-BSP-2	F: GAATAATGTGTGGTTTTTGTTGGAT<br>R: CCACAACCATCAATAATTATATCACATC
ChIP qPCR primers (distal) for NPY1R	F: CTTATCCTGCTGTGAAGAAGC<br>R: CTTCAGGCTTTTCTTCATTGG
ChIP qPCR primers (P1) for NPY1R	F: GAATCAGGTAAAACATCTAAG<br>R: GAACCATGAGCTGAATATTTC
ChIP qPCR primers (P2) for NPY1R	F: TGTTTCCCAGCGAGCCCTTTG<br>R: CCAACAGAAGCCACACATTATTC
