# Supplementary material for: UHRF1 deficiency exacerbates intestinal inflammation by epigenetic modulation of NPY1R gene methylation
Source: JCI Insight. 2026 Feb 9;11(3):e190894. doi: 10.1172/jci.insight.190894 (PMC12892884; doi:10.1172/jci.insight.190894)

Figure 1E

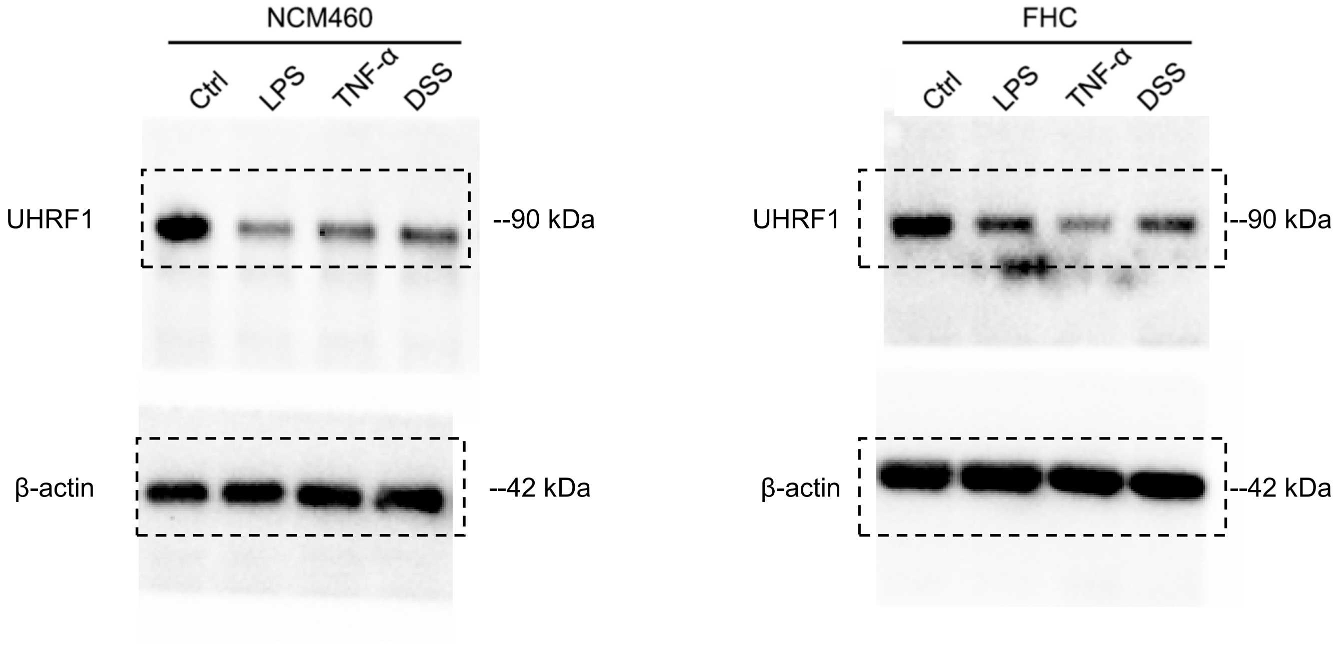

Figure S1E

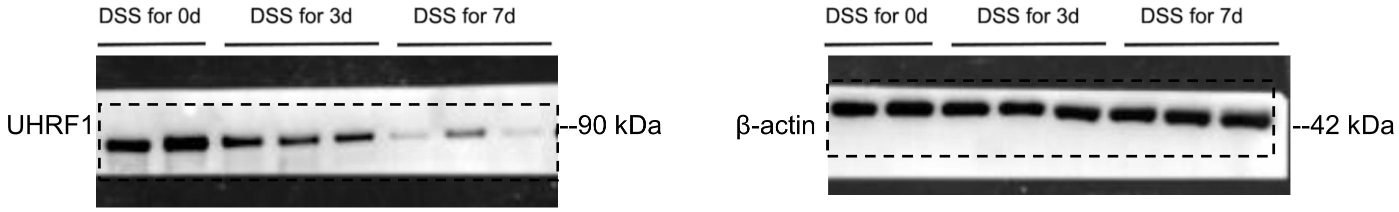

Figure S2A

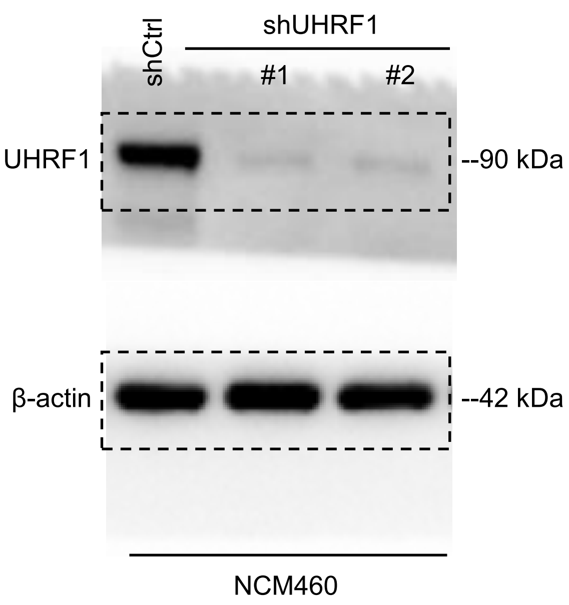

Figure S2B

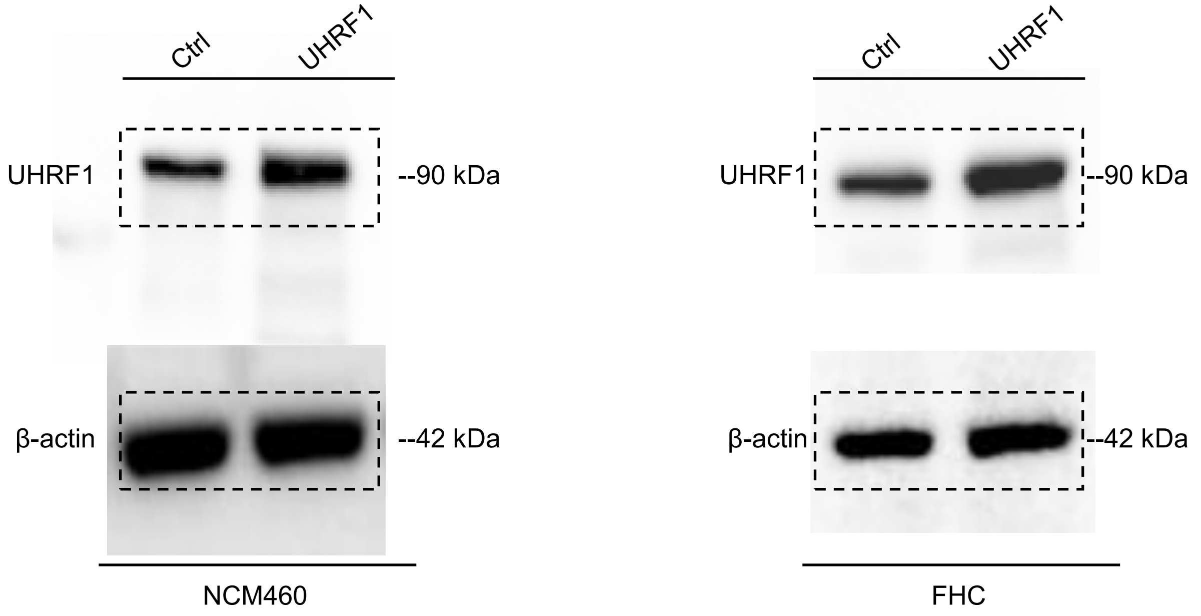

Figure 3G

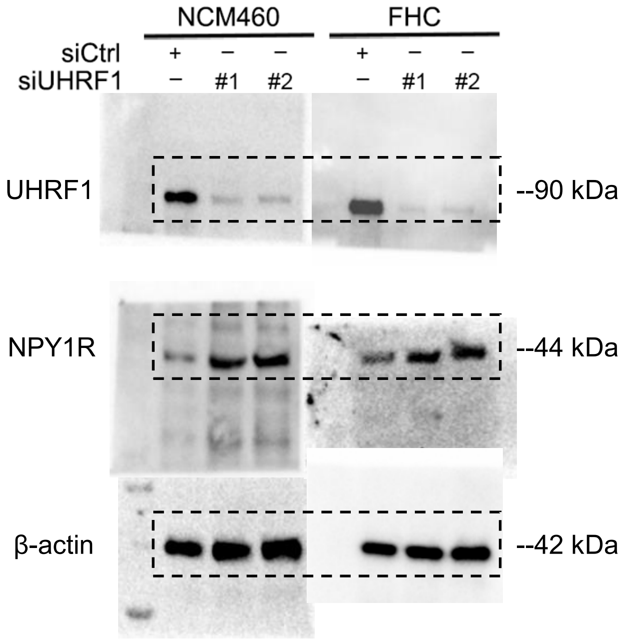

Figure 3H

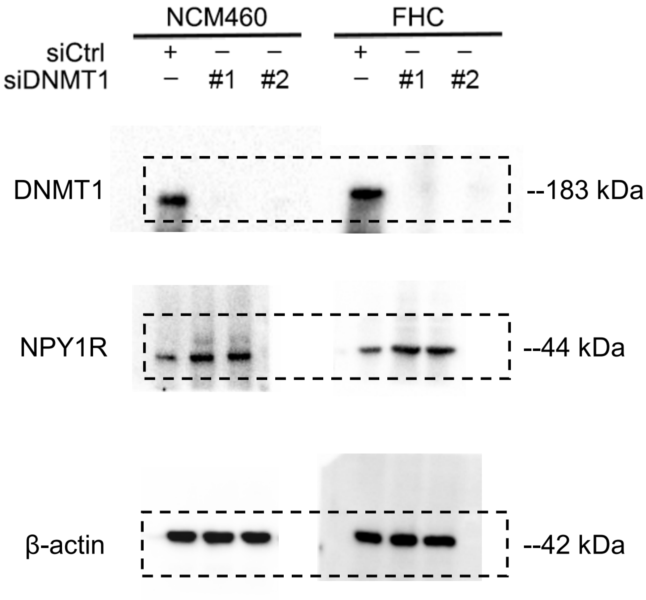

Figure S3B

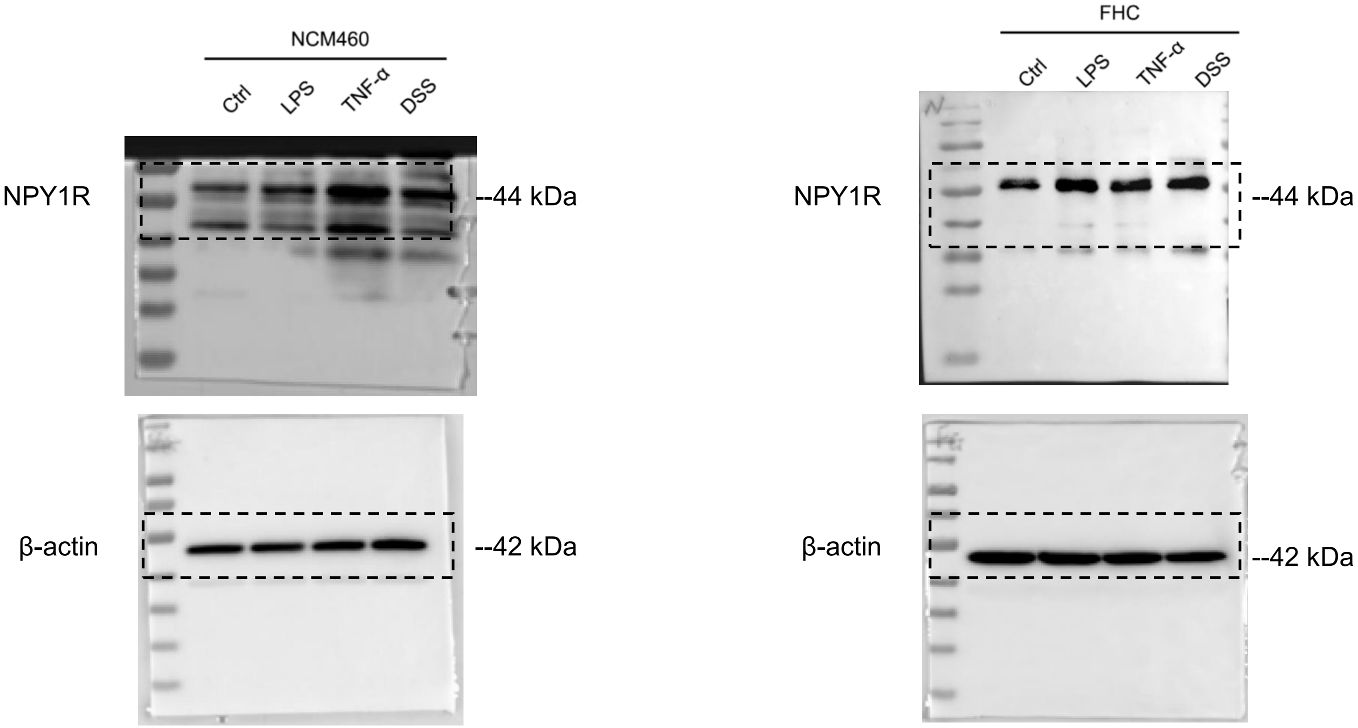

Figure S4A

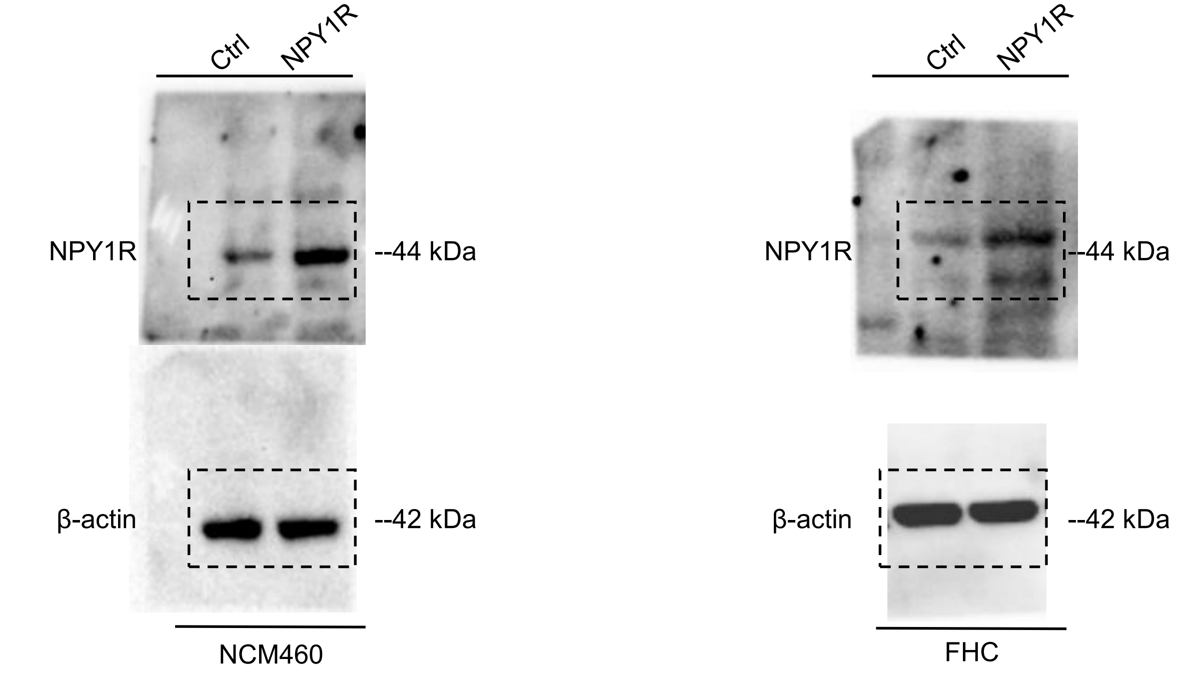

Figure S4B

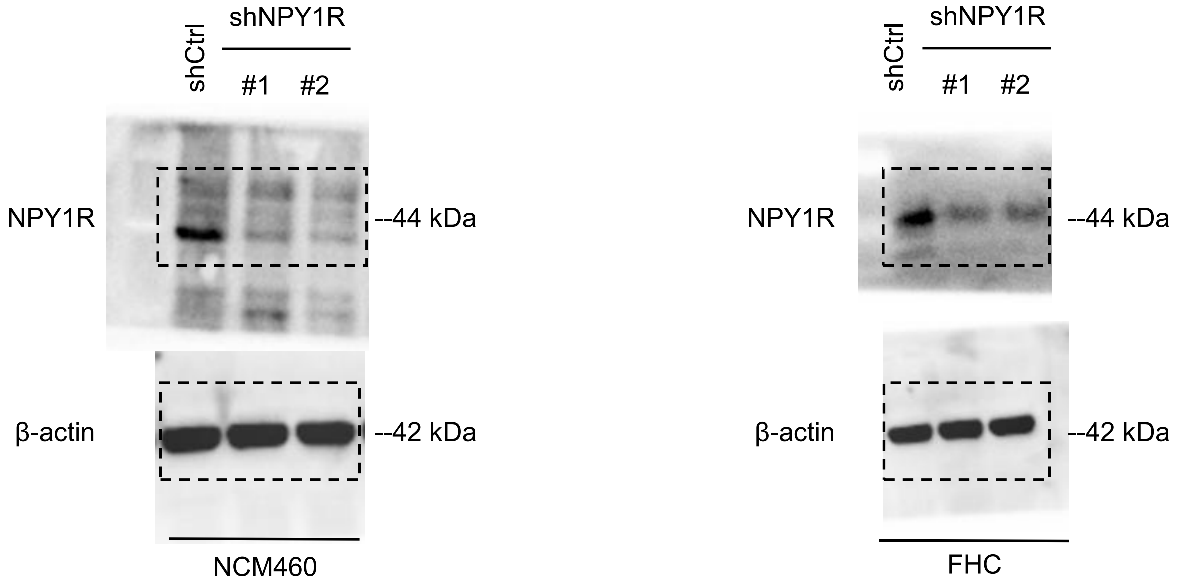

Figure 5A

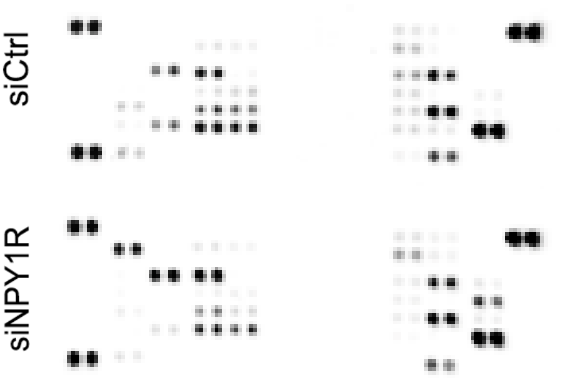

Figure 5B

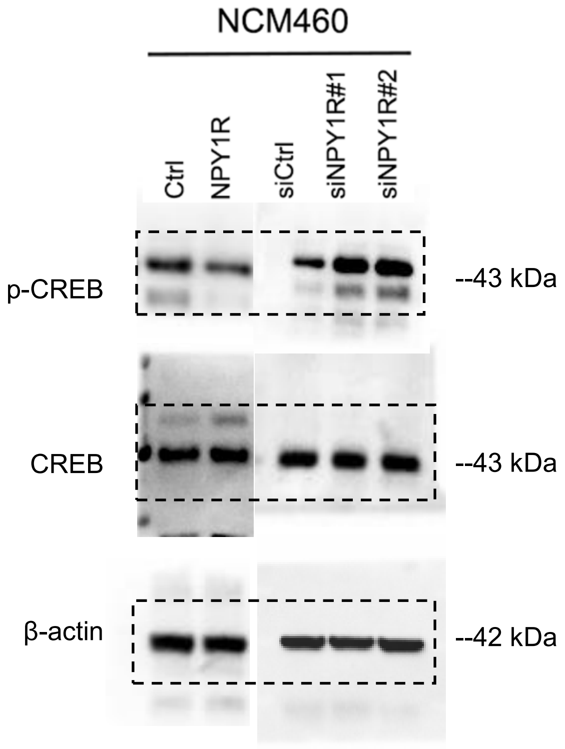

Figure 5C

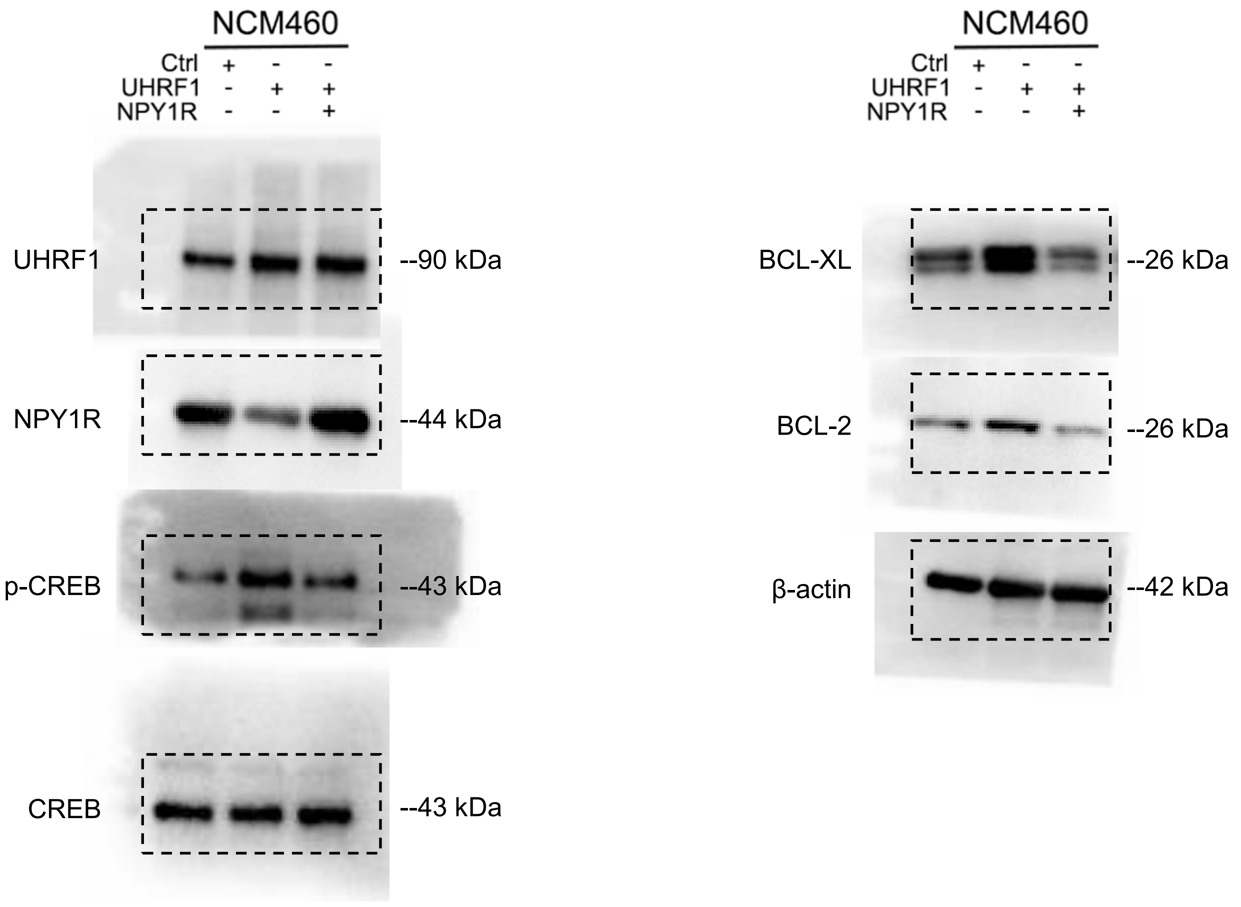

Figure 5D

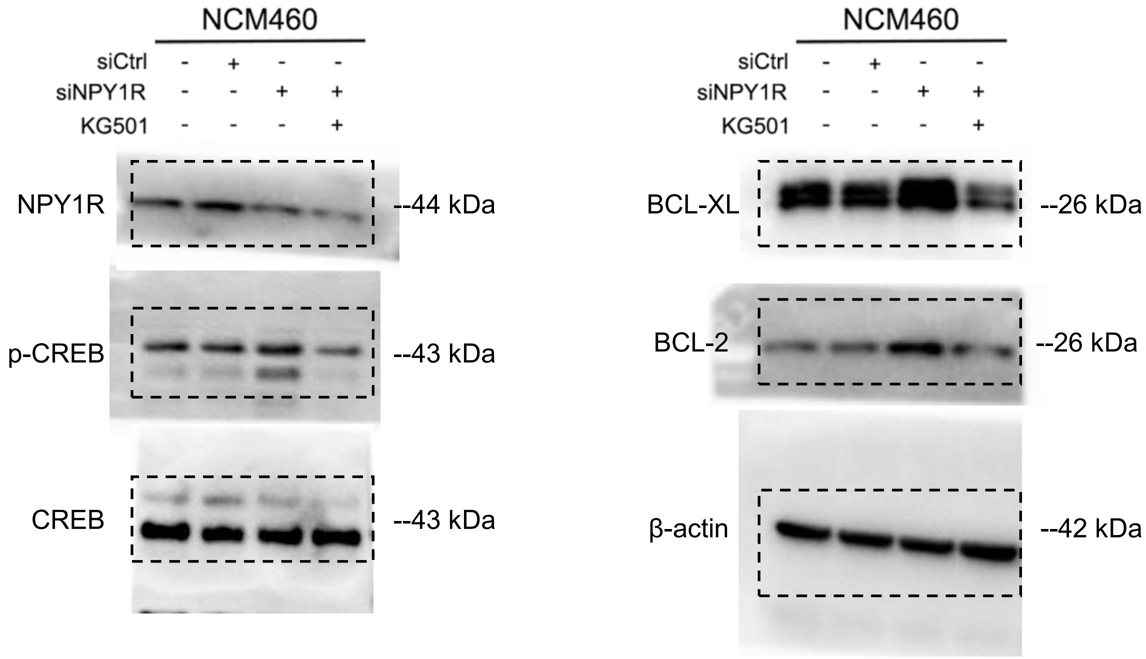
NCM460
siCtrl - + - -
siNPY1R - - + +
KG501 - - - +
NPY1R
--44 kDa
p-CREB
--43 kDa
CREB
--43 kDa
NCM460
siCtrl - + - -
siNPY1R - - + +
KG501 - - - +
BCL-XL
--26 kDa
BCL-2
--26 kDa
β-actin
--42 kDa

Figure 5E

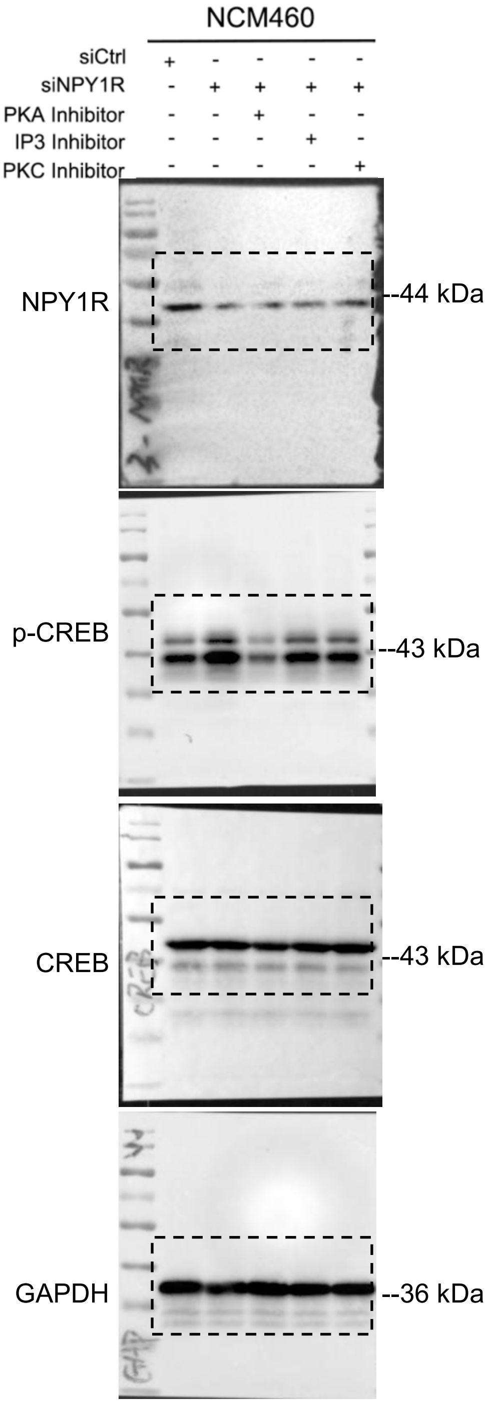

Figure 5G

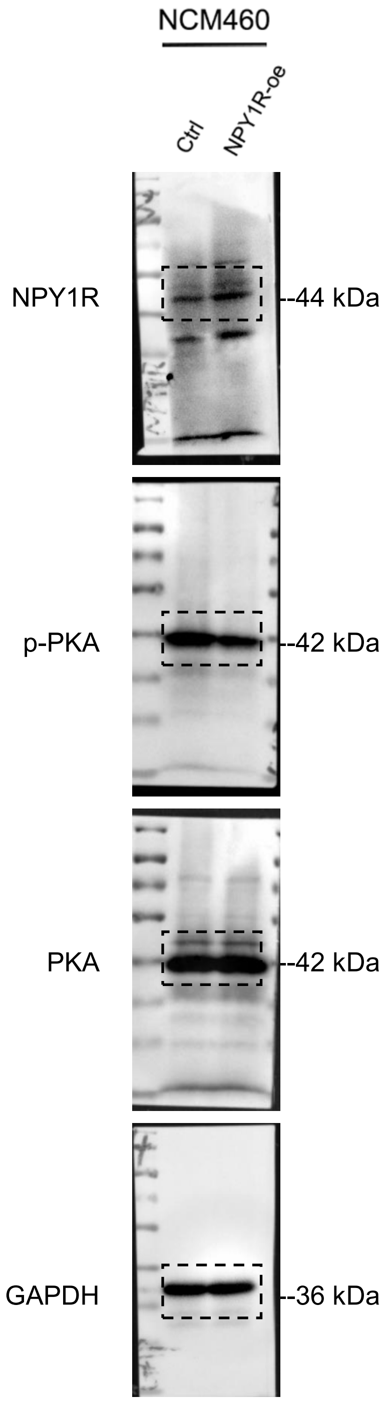

Figure 5I

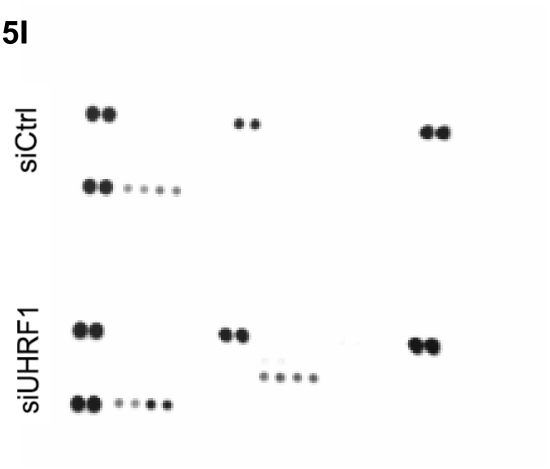

Figure S5A

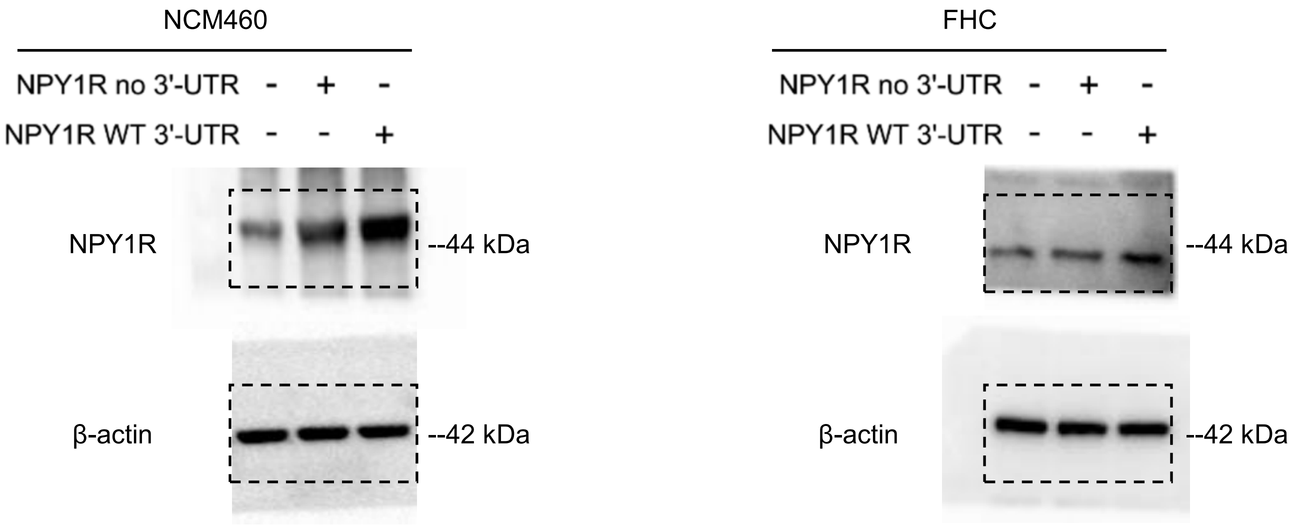

Figure 6D

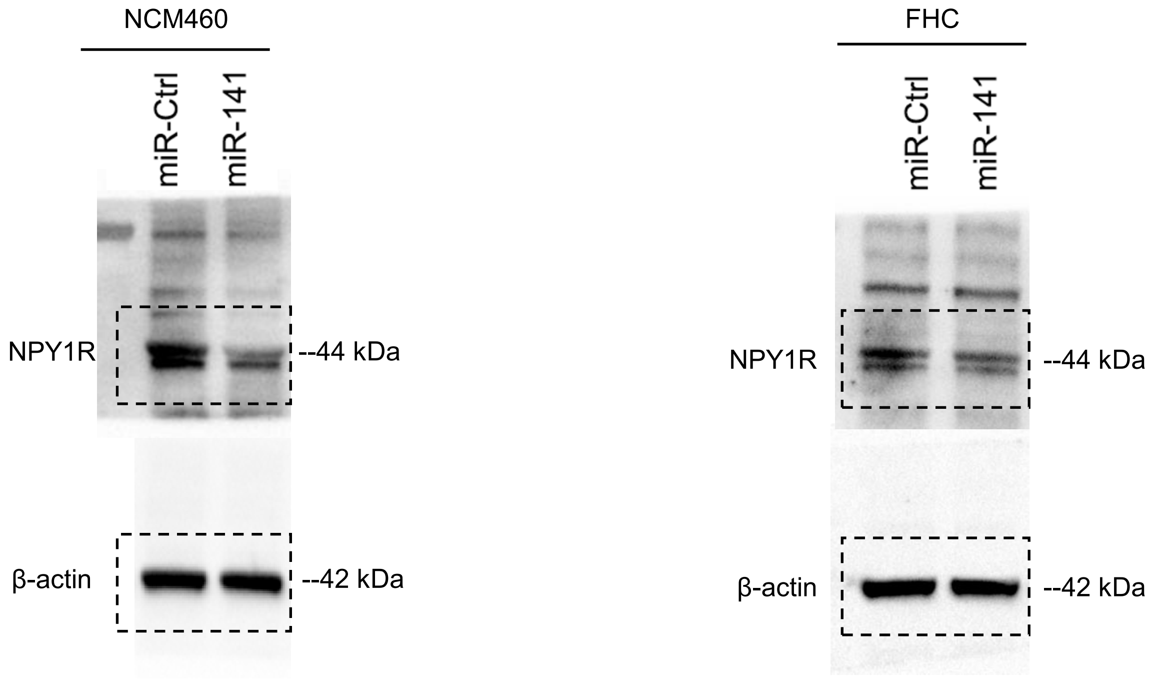

Figure 6F

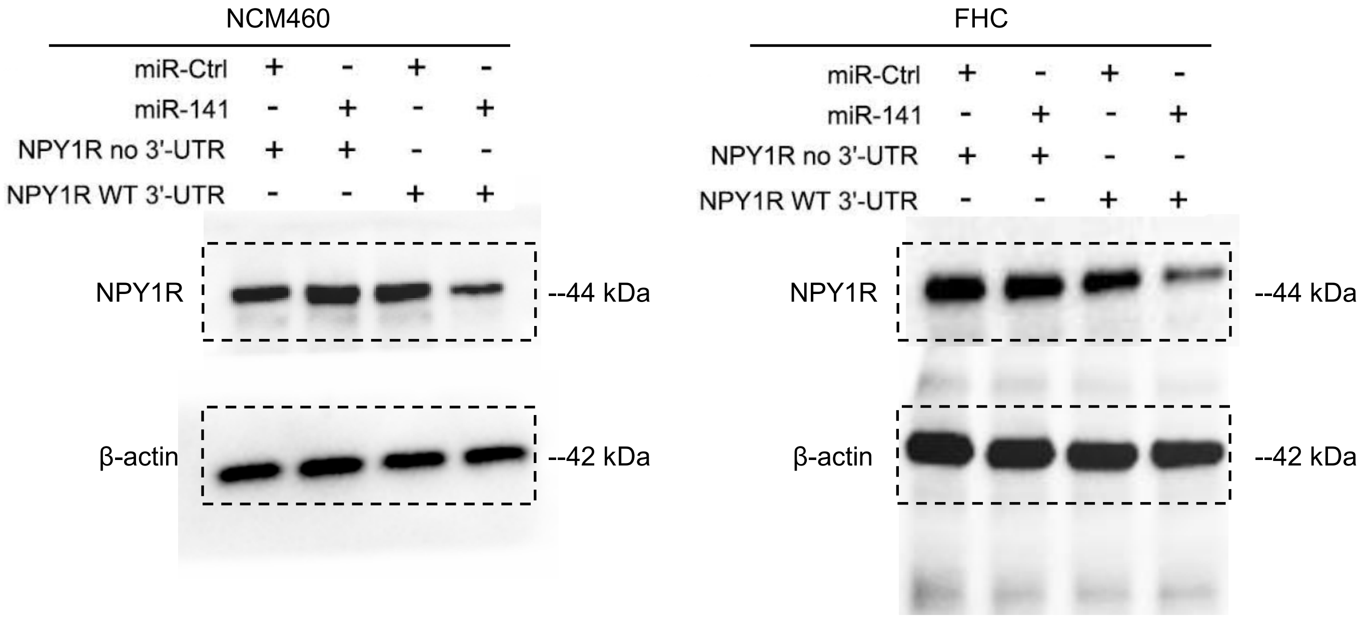

Supplement: Unedited blot and gel images [file jciinsight-11-190894-s019.pdf]
